# Crystal structures of three sterically congested disilanes

**DOI:** 10.1107/S2056989017002602

**Published:** 2017-02-28

**Authors:** Kothanda Rama Pichaandi, Joel T. Mague, Mark J. Fink

**Affiliations:** a Department of Chemistry, Tulane University, New Orleans, LA 70118, USA

**Keywords:** crystal structure, disilane, halogen–halogen inter­action

## Abstract

In the three sterically congested silanes, C_24_H_38_Si_2_ (**1**) (1,1,2,2-tetra­isopropyl-1,2-di­phenyl­disilane), C_24_H_34_Br4Si_2_ (**2**) [1,1,2,2-tetra­kis­(2-bromo­propan-2-yl)-1,2-di­phenyl­disilane] and C_32_H_38_Si_2_ (**3**) (1,2-di-*tert*-butyl-1,1,2,2-tetra­phenyl­disilane), the Si—Si bond length is shortest in (**1**) and longest in (**2**), with (**3**) having an inter­mediate value, which parallels the increasing steric congestion. A comparison of the two isopropyl derivatives, (**1** and **2**), shows a significant increase in the Si—C(*ipso*) distance with the introduction of bromine. Also, in the brominated compound **2**, attractive inter­molecular Br⋯Br inter­actions exist with Br⋯Br separations *ca* 0.52 Å shorter than the sum of the van der Waals radii. In compound **2**, one of the bromo­isopropyl groups is rotationally disordered in an 0.8812 (9):0.1188 (9) ratio. Compound **3** exhibits ‘whole mol­ecule’ disorder in a 0.9645 (7):0.0355 (7) ratio with the Si—Si bonds in the two components making an angle of *ca* 66°.

## Abstract 2

Three sterically congested silanes, namely 1,1,2,2-tetra­isopropyl-1,2-di­phenyl­disilane, 1,1,2,2-tetra­kis­(2-bromo­propan-2-yl)-1,2-di­phenyl­disilane and 1,2-di-*tert*-butyl-1,1,2,2-tetra­phenyl­disilane, show lengthening of the Si—Si and Si—C bonds as compared with disilanes with smaller substituents. The packing of the tetra­kis­(2-bromo­propan-2-yl) compound is partly organized by attractive Br⋯Br inter­actions.

## Chemical context   

The study of tetra­isopropyl- and tetra­kis­(2-bromo­propan-2-yl)-substituted disilanes is of inter­est due to their importance in the synthesis of bis­(silanes), which are precursors for generating transient disilynes (Pichaandi *et al.*, 2011 ▸; Kabe *et al.*, 2000 ▸; Ando *et al.*, 1997 ▸). The synthesis of 1,1,2,2-tetra­isopropyl-1,2-di-*tert*-butyl­disilane and 1,1,2,2-tetra­kis­(2-bromo­propan-2-yl)-1,2-di-*tert*-butyl­disilane were recently reported by our group (Pichaandi *et al.*, 2011 ▸) and the crystal structure of the former determined. However, the structure of the latter could not be solved due to its highly disordered nature, so the exact nature of the influence of the bromine atom in the isopropyl group on the disilane structure could not be determined. We report here a comparison of the structures of 1,1,2,2-tetra­isopropyl-1,2-di­phenyl­disilane (**1**) and 1,1,2,2-tetra­kis­(2-bromo­propan-2-yl)-1,2-di­phenyl­disilane (**2**), as well as that of the related 1,2-di-*tert*-butyl-1,1,2,2-tetra­phenyl­disilane (**3**).
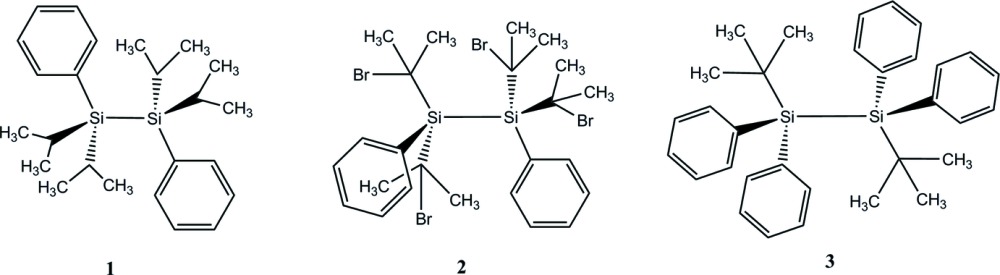


## Structural commentary   

**Figure 1:**
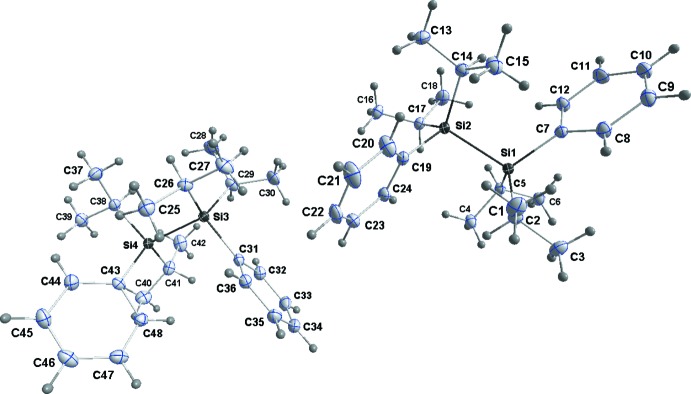
Perspective view of the two independent mol­ecules of **1**, with labeling scheme and 50% probability displacement ellipsoids.

**Figure 2:**
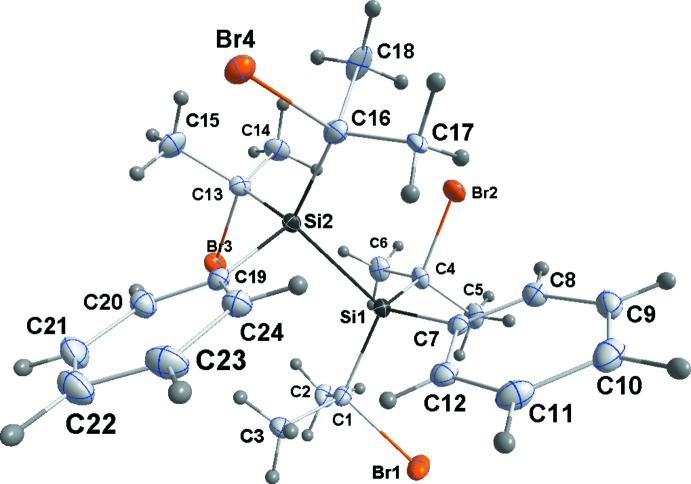
Perspective view of **2**, with labeling scheme and 50% probability displacement ellipsoids. Only the major orientation of the disordered bromo­isopropyl group is shown.

The asymmetric unit for **1** consists of two independent mol­ecules (Fig. 1 ▸), one having an *anti­clinal* conformation and the other a *gauche* conformation about the Si—Si bond. Thus, the torsion angle defined by the Si—Si bond and the *ipso* carbon atoms of the phenyl groups are −140.15 (5)° (C2—Si1—Si2—C19) for the former and 59.58 (6)° (C31—Si3—Si4—C43) for the latter. In contrast, the two independent mol­ecules in the low-temperature form of 1,1,2,2-tetra-*tert-*butyl-1,2-di­phenyl­disilane both adopt the *gauche* arrangement with corresponding torsion angles of −71.47 (9) and −68.61 (9)° (Scholz *et al.*, 2014 ▸). Disilane **2** (Fig. 2 ▸) has a *gauche* conformation with the corresponding torsion angle being 75.55 (5)° (C7—Si1—Si2—C19). The *gauche* conformation in **2** appears to be preferred over other conformations when the rotational barrier around the Si—Si bond is high. This trend is observed in the crowded 1,1,2,2-tetra­isopropyl-1,2-di-*tert*-butyl­disilane (Pichaandi *et al.*, 2011 ▸) and 1,1,2,2-tetra-*tert*-butyl-1,2-di­phenyl­disilane (Lerner *et al.*, 2001 ▸), which both exhibit a *gauche* conformation. However, the sterically less hindered 1,1,2,2-tetra-*tert-*butyl-1,2-di­chloro­disilane (Peters *et al.*, 1998 ▸) and tetra-*tert-*butyl-1,2-di­hydroxy­disilane (West & Pham, 1991 ▸) have an *anti­clinal* conformation, similar to **1**. The higher rotational barrier in **2** comes from the presence of the bulky bromo­isopropyl group.

**Figure 3:**
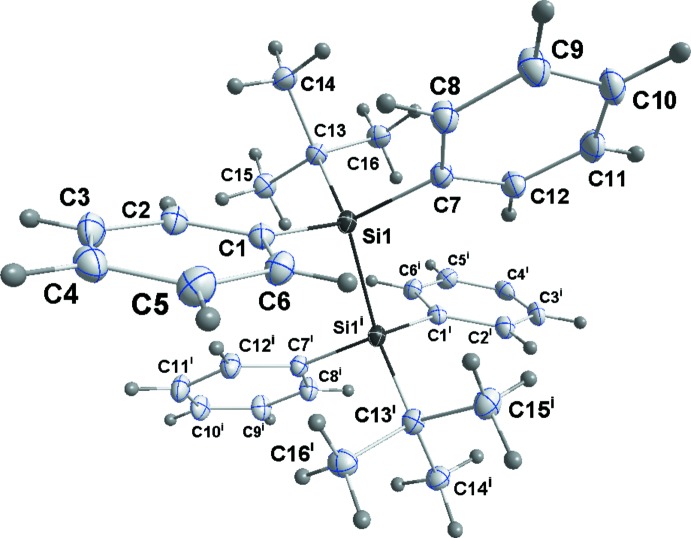
Perspective view of **3**, with labeling scheme and 50% probability displacement ellipsoids. Only the major orientation of the disorder is shown [symmetry code: (i) 2 − *x*, −*y*, −*z*].

Compound **3** has crystallographically imposed centrosymmetry and so adopts a staggered conformation (Fig. 3 ▸). Inter­estingly, in this crystal there is an example of ‘whole mol­ecule’ disorder with 4% of the contents of the asymmetric unit adopting an orientation in which the Si—Si bond is inclined by approximately 66° to that of the major component. Since this work was undertaken, the structure of **3** has been reported by two different groups (Monakhov *et al.*, 2010 ▸; Wei *et al.*, 2014 ▸), but only mentioned cursorily and with no discussion of structural details. The Si—Si bond lengths in **1**–**3** are, respectively, 2.3898 (4), 2.4746 (10) and 2.4002 (6) Å, significantly longer than the typical values for less-congested disilanes, *e.g.* 2.340 (9) Å in hexa­methyl­disilane (Beagley *et al.*, 1971 ▸). The longest compares favorably with those found in the sterically congested disilanes 1,1,2,2-tetra­isopropyl-1,2-di-*tert*-butyl­disilane [2.4787 (6) Å; Pichaandi *et al.*, 2011 ▸] and 1,1,2,2-tetra-*tert-*butyl-1,2-di­phenyl­disilane [2.4973 (8) Å; Lerner *et al.*, 2001 ▸; Scholz *et al.*, 2014 ▸], but is shorter than that in the extremely congested hexa-*tert-*butyl­disilane [2.6863 (5) Å; Scholz *et al.*, 2014 ▸]. The effects of the steric congestion are also seen in the Si—C bond lengths, *e.g.* Si1—C2 = 1.9005 (12) Å in **1**, Si1—C1 = 1.965 (3) Å in **2** and Si1—C13 = 1.9226 (12) Å in **3**, all of which are significantly longer than a typical Si—C single bond (1.87 Å; Sheldrick, 1989 ▸). Additionally, the significant increase in the quoted Si—C bond length between **2** and **1** indicates the increase in steric congestion on brominating the isopropyl group.

## Supra­molecular features   

**Figure 4:**
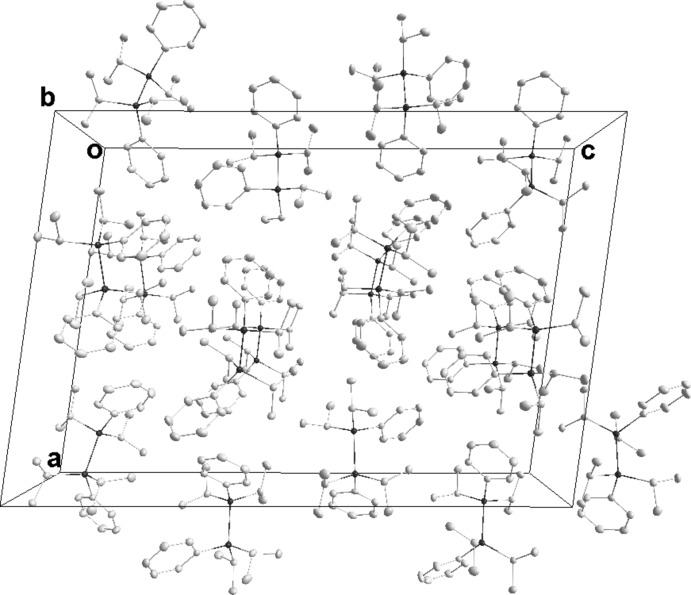
Packing of **1**, viewed along the *b*-axis direction.

**Figure 5:**
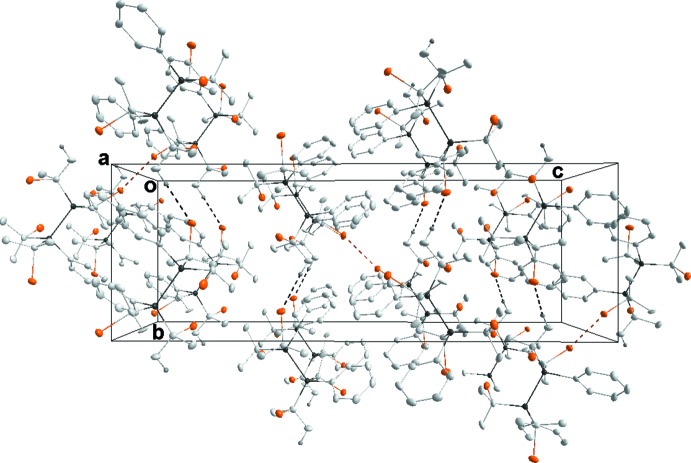
Packing of **2**, viewed along the *a*-axis direction, with the C—H⋯Br hydrogen bonds (Table 1 ▸) shown as black dotted lines and Br⋯Br inter­actions as brown dotted lines.

**Figure 6:**
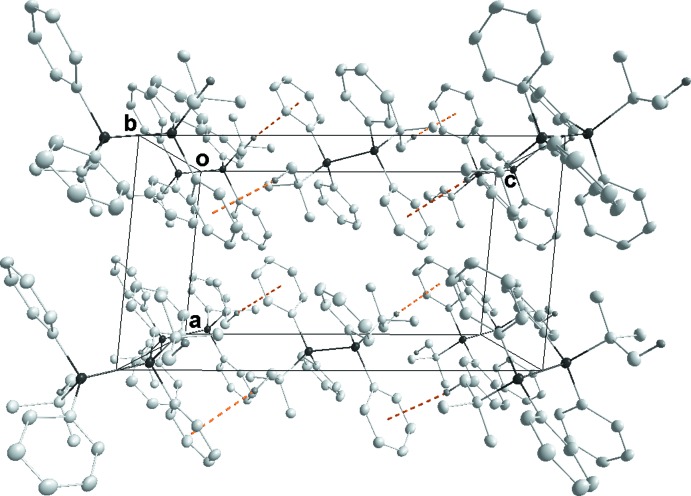
Packing of **3**, viewed along the *b*-axis direction, with the C—H⋯π(ring) inter­actions (Table 2 ▸) shown as dotted lines.

In **1**, the packing consists of layers two mol­ecules thick which are parallel to (001) with only normal van der Waals contacts between mol­ecules (Fig. 4 ▸). In **2**, the mol­ecules form chains running parallel to the *b*-axis direction through weak C—H⋯Br hydrogen bonds (see Table 1 ▸). These chains pair up through Br4⋯Br4 (−*x* + 1, −*y* + 1, −*z* + 1) inter­actions, where the Br⋯Br separation of 3.1755 (7) Å is 0.52 Å shorter than the sum of the van der Waals radii (3.70 Å) (see Fig. 5 ▸). We consider these to be attractive inter­actions as has been argued previously (Desiraju & Parthasarthy, 1989 ▸). Only normal van der Waals contacts occur between the double chains. The primary inter­molecular inter­action in **3** is a C—H⋯π inter­action (see Table 2 ▸), which forms chains running parallel to the *c*-axis direction (Fig. 6 ▸).

## Database survey   

**Figure 7:**
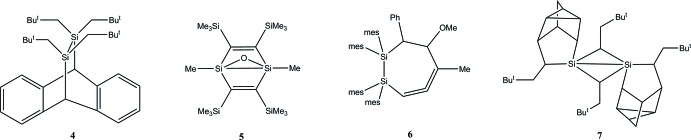
Compounds from the database survey.

There are 390 structures of disilanes containing only Si—C bonds to the substituents in the Cambridge Crystallographic Database (CSD, V5.38, last update November, 2016; Groom *et al.*, 2016 ▸), but in only 43 of these is the Si—Si distance greater than 2.40 Å. In this set, the distances range from 2.401 (2) Å in **4** (Kyushin *et al.*, 1996 ▸) (Fig. 7 ▸). to 2.6863 (5) Å in one structure of hexa-*tert-*butyl­disilane (Scholz *et al.*, 2014 ▸). In addition to the four reported structures of hexa-*tert-*butyl­disilane (Scholz *et al.*, 2012 ▸, 2014 ▸; Wiberg *et al.*, 1986 ▸; Wiberg & Niedermayer, 2000 ▸), but excluding the five examples where it is co-crystallized with [NaO*R*]_4_ (Lerner *et al.*, 2002 ▸), [Sn*R*]_6_ (Wiberg *et al.*, 1999 ▸), [Si*R*]_4_ (Wiberg *et al.*, 1993 ▸; Meyer-Wegner *et al.*, 2009 ▸) and [Ge*R*]_4_ (Wiberg *et al.*, 1996 ▸) [*R* = Si(*t*-Bu)_3_ in all cases], only four other mol­ecules have Si—Si distances greater than 2.5 Å. These are **5** [2.5149 (13) Å; Kabe *et al.*, 2000 ▸), Ph_6_Si_2_ as a solid solution with Ph_6_Pb_2_ [2.519 (4) Å; Kleiner & Dräger, 1984 ▸], **6** [2.5428 (18) Å; Gottschling *et al.*, 2005 ▸] and **7** [2.6468 (9) Å; Goetze *et al.*, 1997 ▸] (Fig. 7 ▸).

## Synthesis and crystallization   

Disilanes **1** and **2** were prepared according to the literature procedures (Lambert & Urdaneta-Perez, 1978 ▸; Pichaandi *et al.*, 2011 ▸). Colorless crystals of **1** and **2** were obtained from hexane and di­chloro­methane solutions, respectively. To prepare **3**, a 50 ml Schlenk flask was loaded with *tert*-butyl­diphenyl­chloro­silane (10 g, 37 mmol), finely cut Li wire (0.26 g, 0.038 g-atom) and 20 ml of THF under nitro­gen and the mixture was stirred overnight at 298 K. The reaction mixture was then diluted with 350 ml of CH_2_Cl_2_ and dilute HCl (10 ml) and 20 ml of water were added. The organic layer was then separated from the aqueous layer, dried with MgSO_4_ and the solvent removed *in vacuo* to give **3** as a white powder. Crystals suitable for X-ray diffraction were obtained from CH_2_Cl_2_ solution (yield 8.1 g, 94%). ^1^H NMR (δ, CD_2_Cl_2_) 0.76–1.02 (*s*, 18H) 7.27–7.52 (*m*, 12H) 7.65–7.85 (*m*, 8H); ^13^C{^1^H} NMR (δ, CD_2_Cl_2_) 20.0, 28.8, 127.8, 128.9, 136.6, 137.5; ^29^Si{^1^H} NMR (δ, CD_2_Cl_2_) −13.5.

## Refinement   

Crystal data, data collection and structure refinement details are summarized in Table 3 ▸. In compound **2**, the bromo­isopropyl group containing Br4 is rotationally disordered about the Si2—C16 axis in an 0.8812 (9):0.1188 (9) ratio. The two components of the disorder were refined with restraints that their geometries be comparable to one another and to those of the other three bromo­isopropyl groups. Compound **3** exhibits ‘whole mol­ecule’ disorder in a 0.9645 (7):0.0355 (7) ratio with the Si—Si bonds in the two components making an angle of *ca* 66°. The alternate location of the unique Si atom was obtained from a difference Fourier map and its inclusion in the structure-factor calculation allowed enough atoms of its phenyl groups to be located so that these could be completed and refined as rigid hexa­gons. Following this, the remaining atoms of the minor component could be located and they were refined with restraints that the geometry be comparable with that of the major component. In all three structures, the H atoms were included as riding contributions in idealized positions: C—H = 0.95–0.98 Å with *U*_iso_(H) = 1.5*U*_eq_(C-meth­yl) and 1.2*U*_eq_(C) for other H atoms.

## Supplementary Material

Crystal structure: contains datablock(s) 1, 2, 3, global. DOI: 10.1107/S2056989017002602/su5352sup1.cif

Structure factors: contains datablock(s) 1. DOI: 10.1107/S2056989017002602/su53521sup2.hkl

Structure factors: contains datablock(s) 2. DOI: 10.1107/S2056989017002602/su53522sup3.hkl

Structure factors: contains datablock(s) 3. DOI: 10.1107/S2056989017002602/su53523sup4.hkl

Supporting information file. DOI: 10.1107/S2056989017002602/su53521sup5.cml

Supporting information file. DOI: 10.1107/S2056989017002602/su53522sup6.cml

Supporting information file. DOI: 10.1107/S2056989017002602/su53523sup7.cml

CCDC references: 1532770, 1532769, 1532768

Additional supporting information:  crystallographic information; 3D view; checkCIF report

## Figures and Tables

**Table 1:** Hydrogen-bond geometry (Å, °) for **2** [Chem scheme1]

*D*—H⋯*A*	*D*—H	H⋯*A*	*D*⋯*A*	*D*—H⋯*A*
C15—H15*C*⋯Br1^i^	0.98	2.82	3.771 (3)	166

Symmetry code: (i) 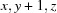.

**Table 2:** Hydrogen-bond geometry (Å, °) for **3** [Chem scheme1] *Cg*1 is the centroid of C1–C6 the ring.

*D*—H⋯*A*	*D*—H	H⋯*A*	*D*⋯*A*	*D*—H⋯*A*
C15—H15*C*⋯*Cg*1^i^	0.98	2.93	3.8955 (14)	171

Symmetry code: (i) 
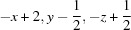
.

**Table 3:** Experimental details

	**1**	**2**	**3**
Crystal data			
Chemical formula	C_24_H_38_Si_2_	C_24_H_34_Br_4_Si_2_	C_32_H_38_Si_2_
*M*_r_	382.72	698.33	478.80
Crystal system, space group	Monoclinic, *P*2_1_/*n*	Monoclinic, *P*2_1_/*c*	Monoclinic, *P*2_1_/*c*
Temperature (K)	100	100	100
*a*, *b*, *c* (Å)	19.8418 (14), 8.2554 (6), 28.454 (2)	8.8779 (7), 10.4042 (8), 29.699 (2)	8.5622 (5), 10.2107 (6), 15.4586 (10)
β (°)	97.838 (1)	90.975 (1)	95.452 (1)
*V* (Å^3^)	4617.3 (6)	2742.8 (4)	1345.37 (14)
*Z*	8	4	2
Radiation type	Mo *K*α	Mo *K*α	Mo *K*α
μ (mm^−1^)	0.16	5.97	0.15
Crystal size (mm)	0.22 × 0.19 × 0.14	0.14 × 0.12 × 0.07	0.17 × 0.15 × 0.13

Data collection			
Diffractometer	Bruker SMART APEX CCD	Bruker SMART APEX CCD	Bruker SMART APEX CCD
Absorption correction	Multi-scan (*SADABS*; Bruker, 2016 ▸)	Multi-scan (*SADABS*; Bruker, 2016 ▸)	Multi-scan (*SADABS*; Bruker, 2016 ▸)
*T*_min_, *T*_max_	0.96, 0.98	0.49, 0.69	0.98, 0.98
No. of measured, independent and observed [*I* > 2σ(*I*)] reflections	81051, 12375, 10320	47392, 6875, 5471	23513, 3566, 3065
*R*_int_	0.046	0.052	0.031
(sin θ/λ)_max_ (Å^−1^)	0.696	0.669	0.693

Refinement			
*R*[*F* ^2^ > 2σ(*F* ^2^)], *wR*(*F* ^2^), *S*	0.036, 0.096, 1.03	0.028, 0.062, 1.03	0.034, 0.092, 1.06
No. of reflections	12375	6875	3566
No. of parameters	485	287	179
No. of restraints	0	38	43
H-atom treatment	H-atom parameters constrained	H-atom parameters constrained	H-atom parameters constrained
Δρ_max_, Δρ_min_ (e Å^−3^)	0.39, −0.29	1.00, −1.05	0.38, −0.29

Computer programs: *APEX3* and *SAINT* (Bruker, 2016 ▸), *SHELXT* (Sheldrick, 2015*a* ▸), *SHELXL2014* (Sheldrick, 2015*b* ▸), *DIAMOND* (Brandenburg & Putz, 2012 ▸) and *SHELXTL* (Sheldrick, 2008 ▸).

## supplementary crystallographic information

### (1) 1,1,2,2-Tetraisopropyl-1,2-diphenyldisilane Crystal data

**Table:**

C_24_H_38_Si_2_	*F*(000) = 1680
*M**_r_* = 382.72	*D*_x_ = 1.101 Mg m^−^^3^
Monoclinic, *P*2_1_/*n*	Mo *K*α radiation, λ = 0.71073 Å
*a* = 19.8418 (14) Å	Cell parameters from 9612 reflections
*b* = 8.2554 (6) Å	θ = 2.4–29.4°
*c* = 28.454 (2) Å	µ = 0.16 mm^−^^1^
β = 97.838 (1)°	*T* = 100 K
*V* = 4617.3 (6) Å^3^	Block, colorless
*Z* = 8	0.22 × 0.19 × 0.14 mm

### (1) 1,1,2,2-Tetraisopropyl-1,2-diphenyldisilane Data collection

**Table:**

Bruker SMART APEX CCD diffractometer	12375 independent reflections
Radiation source: fine-focus sealed tube	10320 reflections with *I* > 2σ(*I*)
Graphite monochromator	*R*_int_ = 0.046
φ and ω scans	θ_max_ = 29.7°, θ_min_ = 2.1°
Absorption correction: multi-scan (*SADABS*; Bruker, 2016)	*h* = −27→27
*T*_min_ = 0.96, *T*_max_ = 0.98	*k* = −11→11
81051 measured reflections	*l* = −39→38

### (1) 1,1,2,2-Tetraisopropyl-1,2-diphenyldisilane Refinement

**Table:**

Refinement on *F*^2^	Primary atom site location: structure-invariant direct methods
Least-squares matrix: full	Secondary atom site location: difference Fourier map
*R*[*F*^2^ > 2σ(*F*^2^)] = 0.036	Hydrogen site location: inferred from neighbouring sites
*wR*(*F*^2^) = 0.096	H-atom parameters constrained
*S* = 1.03	*w* = 1/[σ^2^(*F*_o_^2^) + (0.0424*P*)^2^ + 1.8169*P*] where *P* = (*F*_o_^2^ + 2*F*_c_^2^)/3
12375 reflections	(Δ/σ)_max_ = 0.002
485 parameters	Δρ_max_ = 0.39 e Å^−^^3^
0 restraints	Δρ_min_ = −0.29 e Å^−^^3^

### (1) 1,1,2,2-Tetraisopropyl-1,2-diphenyldisilane Special details

**Table:**

Experimental. The diffraction data were obtained from 3 sets of 400 frames, each of width 0.5° in ω, colllected at φ = 0.00, 90.00 and 180.00° and 2 sets of 800 frames, each of width 0.45° in φ, collected at ω = –30.00 and 210.00°. The scan time was 15 sec/frame.
Geometry. All esds (except the esd in the dihedral angle between two l.s. planes) are estimated using the full covariance matrix. The cell esds are taken into account individually in the estimation of esds in distances, angles and torsion angles; correlations between esds in cell parameters are only used when they are defined by crystal symmetry. An approximate (isotropic) treatment of cell esds is used for estimating esds involving l.s. planes.
Refinement. Refinement of F^2^ against ALL reflections. The weighted R-factor wR and goodness of fit S are based on F^2^, conventional R-factors R are based on F, with F set to zero for negative F^2^. The threshold expression of F^2^ > 2sigma(F^2^) is used only for calculating R-factors(gt) etc. and is not relevant to the choice of reflections for refinement. R-factors based on F^2^ are statistically about twice as large as those based on F, and R- factors based on ALL data will be even larger. H-atoms attached to carbon were placed in calculated positions (C—H = 0.95 - 1.00 Å). All were included as riding contributions with isotropic displacement parameters 1.2 - 1.5 times those of the attached atoms.

### (1) 1,1,2,2-Tetraisopropyl-1,2-diphenyldisilane Fractional atomic coordinates and isotropic or equivalent isotropic displacement parameters (Å^2^)

**Table:**

	*x*	*y*	*z*	*U*_iso_*/*U*_eq_	
Si1	0.94710 (2)	0.33575 (4)	0.11131 (2)	0.01283 (7)	
Si2	0.84650 (2)	0.49225 (4)	0.11451 (2)	0.01282 (7)	
C1	0.94625 (7)	0.22143 (17)	0.20748 (4)	0.0246 (3)	
H1A	0.9414	0.1265	0.2274	0.037*	
H1B	0.9060	0.2905	0.2068	0.037*	
H1C	0.9868	0.2827	0.2205	0.037*	
C2	0.95337 (6)	0.16598 (14)	0.15680 (4)	0.0182 (2)	
H2	0.9133	0.0944	0.1469	0.022*	
C3	1.01631 (7)	0.05704 (16)	0.15684 (5)	0.0246 (3)	
H3A	1.0576	0.1232	0.1628	0.037*	
H3B	1.0146	0.0036	0.1259	0.037*	
H3C	1.0168	−0.0251	0.1818	0.037*	
C4	0.89382 (6)	0.09978 (15)	0.04237 (4)	0.0211 (2)	
H4A	0.8897	0.0626	0.0094	0.032*	
H4B	0.8490	0.1325	0.0498	0.032*	
H4C	0.9116	0.0117	0.0636	0.032*	
C5	0.94264 (6)	0.24497 (14)	0.04919 (4)	0.0153 (2)	
H5	0.9232	0.3305	0.0264	0.018*	
C6	1.01195 (6)	0.19787 (15)	0.03505 (4)	0.0197 (2)	
H6A	1.0315	0.1089	0.0553	0.030*	
H6B	1.0426	0.2915	0.0390	0.030*	
H6C	1.0060	0.1632	0.0018	0.030*	
C7	1.02387 (6)	0.47398 (14)	0.12040 (4)	0.0155 (2)	
C8	1.07141 (6)	0.47422 (15)	0.16149 (4)	0.0192 (2)	
H8	1.0659	0.4006	0.1863	0.023*	
C9	1.12668 (6)	0.57976 (16)	0.16690 (5)	0.0228 (3)	
H9	1.1581	0.5779	0.1953	0.027*	
C10	1.13605 (6)	0.68737 (16)	0.13106 (5)	0.0235 (3)	
H10	1.1738	0.7591	0.1347	0.028*	
C11	1.08994 (7)	0.68980 (16)	0.08981 (5)	0.0247 (3)	
H11	1.0961	0.7631	0.0650	0.030*	
C12	1.03465 (6)	0.58479 (15)	0.08477 (4)	0.0207 (2)	
H12	1.0033	0.5881	0.0564	0.025*	
C13	0.81154 (7)	0.81277 (15)	0.14411 (5)	0.0250 (3)	
H13A	0.7766	0.7606	0.1601	0.037*	
H13B	0.7922	0.8414	0.1116	0.037*	
H13C	0.8276	0.9111	0.1614	0.037*	
C14	0.87141 (6)	0.69571 (14)	0.14283 (4)	0.0180 (2)	
H14	0.9017	0.7480	0.1219	0.022*	
C15	0.91391 (7)	0.68311 (16)	0.19195 (4)	0.0233 (3)	
H15A	0.9278	0.7917	0.2033	0.035*	
H15B	0.9544	0.6171	0.1898	0.035*	
H15C	0.8867	0.6324	0.2142	0.035*	
C16	0.72602 (6)	0.57119 (16)	0.04877 (4)	0.0218 (2)	
H16A	0.7231	0.6757	0.0647	0.033*	
H16B	0.7010	0.4892	0.0643	0.033*	
H16C	0.7061	0.5811	0.0154	0.033*	
C17	0.80103 (6)	0.52024 (14)	0.05162 (4)	0.0163 (2)	
H17	0.8013	0.4123	0.0357	0.020*	
C18	0.83872 (7)	0.63765 (17)	0.02249 (5)	0.0252 (3)	
H18A	0.8181	0.6341	−0.0108	0.038*	
H18B	0.8867	0.6060	0.0249	0.038*	
H18C	0.8355	0.7479	0.0348	0.038*	
C19	0.78538 (6)	0.37687 (14)	0.14769 (4)	0.0164 (2)	
C20	0.76958 (7)	0.41958 (18)	0.19242 (5)	0.0263 (3)	
H20	0.7888	0.5151	0.2073	0.032*	
C21	0.72620 (7)	0.3252 (2)	0.21575 (5)	0.0330 (3)	
H21	0.7168	0.3558	0.2464	0.040*	
C22	0.69685 (7)	0.18726 (18)	0.19442 (5)	0.0274 (3)	
H22	0.6679	0.1220	0.2106	0.033*	
C23	0.70962 (7)	0.14443 (16)	0.14959 (5)	0.0241 (3)	
H23	0.6884	0.0516	0.1344	0.029*	
C24	0.75370 (6)	0.23788 (15)	0.12678 (5)	0.0206 (2)	
H24	0.7626	0.2065	0.0961	0.025*	
Si3	0.44829 (2)	0.13947 (4)	0.11813 (2)	0.01413 (7)	
Si4	0.33848 (2)	0.01815 (4)	0.10635 (2)	0.01342 (7)	
C25	0.43042 (7)	0.20987 (16)	0.21584 (4)	0.0218 (2)	
H25A	0.4572	0.1139	0.2266	0.033*	
H25B	0.3824	0.1796	0.2081	0.033*	
H25C	0.4351	0.2916	0.2411	0.033*	
C26	0.45634 (6)	0.27984 (14)	0.17149 (4)	0.0185 (2)	
H26	0.4259	0.3739	0.1616	0.022*	
C27	0.52755 (7)	0.35206 (17)	0.18431 (5)	0.0270 (3)	
H27A	0.5271	0.4307	0.2101	0.041*	
H27B	0.5412	0.4063	0.1565	0.041*	
H27C	0.5600	0.2653	0.1946	0.041*	
C28	0.42502 (7)	0.41899 (17)	0.05920 (5)	0.0300 (3)	
H28A	0.4446	0.4922	0.0845	0.045*	
H28B	0.3771	0.3985	0.0622	0.045*	
H28C	0.4282	0.4688	0.0283	0.045*	
C29	0.46428 (6)	0.25840 (15)	0.06313 (4)	0.0212 (2)	
H29	0.4451	0.1918	0.0351	0.025*	
C30	0.53954 (7)	0.28767 (18)	0.05824 (5)	0.0301 (3)	
H30A	0.5430	0.3365	0.0273	0.045*	
H30B	0.5640	0.1843	0.0609	0.045*	
H30C	0.5596	0.3609	0.0834	0.045*	
C31	0.51227 (6)	−0.03158 (14)	0.12520 (4)	0.0164 (2)	
C32	0.52628 (6)	−0.11673 (15)	0.08469 (4)	0.0206 (2)	
H32	0.5030	−0.0873	0.0545	0.025*	
C33	0.57320 (7)	−0.24251 (16)	0.08779 (5)	0.0238 (3)	
H33	0.5816	−0.2977	0.0599	0.029*	
C34	0.60787 (6)	−0.28779 (16)	0.13150 (5)	0.0237 (3)	
H34	0.6402	−0.3733	0.1336	0.028*	
C35	0.59481 (6)	−0.20691 (15)	0.17197 (5)	0.0225 (3)	
H35	0.6182	−0.2375	0.2020	0.027*	
C36	0.54761 (6)	−0.08103 (15)	0.16883 (4)	0.0190 (2)	
H36	0.5392	−0.0274	0.1970	0.023*	
C37	0.27046 (7)	0.31327 (16)	0.13141 (5)	0.0252 (3)	
H37A	0.2352	0.3932	0.1207	0.038*	
H37B	0.3152	0.3653	0.1345	0.038*	
H37C	0.2627	0.2695	0.1622	0.038*	
C38	0.26779 (6)	0.17514 (14)	0.09508 (4)	0.0177 (2)	
H38	0.2743	0.2282	0.0644	0.021*	
C39	0.19554 (6)	0.10389 (16)	0.08694 (5)	0.0237 (3)	
H39A	0.1863	0.0475	0.1157	0.035*	
H39B	0.1919	0.0272	0.0604	0.035*	
H39C	0.1625	0.1915	0.0795	0.035*	
C40	0.28249 (7)	−0.26623 (17)	0.05571 (5)	0.0276 (3)	
H40A	0.2362	−0.2221	0.0514	0.041*	
H40B	0.2914	−0.3210	0.0865	0.041*	
H40C	0.2872	−0.3439	0.0303	0.041*	
C41	0.33377 (6)	−0.12780 (16)	0.05397 (4)	0.0203 (2)	
H41	0.3796	−0.1794	0.0556	0.024*	
C42	0.32034 (7)	−0.0415 (2)	0.00566 (4)	0.0298 (3)	
H42A	0.3226	−0.1205	−0.0198	0.045*	
H42B	0.3548	0.0425	0.0040	0.045*	
H42C	0.2751	0.0083	0.0020	0.045*	
C43	0.33233 (6)	−0.10919 (14)	0.16082 (4)	0.0156 (2)	
C44	0.28518 (6)	−0.08099 (16)	0.19235 (4)	0.0202 (2)	
H44	0.2534	0.0050	0.1860	0.024*	
C45	0.28362 (7)	−0.17546 (17)	0.23271 (4)	0.0246 (3)	
H45	0.2507	−0.1547	0.2532	0.030*	
C46	0.33015 (7)	−0.29972 (17)	0.24291 (5)	0.0254 (3)	
H46	0.3297	−0.3633	0.2707	0.031*	
C47	0.37739 (7)	−0.33090 (15)	0.21243 (5)	0.0239 (3)	
H47	0.4093	−0.4163	0.2193	0.029*	
C48	0.37817 (6)	−0.23746 (14)	0.17185 (4)	0.0194 (2)	
H48	0.4105	−0.2610	0.1511	0.023*	

### (1) 1,1,2,2-Tetraisopropyl-1,2-diphenyldisilane Atomic displacement parameters (Å^2^)

**Table:**

	*U*^11^	*U*^22^	*U*^33^	*U*^12^	*U*^13^	*U*^23^
Si1	0.01238 (14)	0.01453 (14)	0.01153 (14)	−0.00076 (11)	0.00148 (11)	0.00086 (11)
Si2	0.01241 (15)	0.01469 (14)	0.01148 (14)	−0.00064 (11)	0.00200 (11)	−0.00024 (11)
C1	0.0270 (7)	0.0314 (7)	0.0158 (6)	0.0038 (5)	0.0040 (5)	0.0076 (5)
C2	0.0184 (6)	0.0185 (5)	0.0176 (5)	−0.0008 (4)	0.0020 (4)	0.0040 (4)
C3	0.0274 (7)	0.0220 (6)	0.0241 (6)	0.0061 (5)	0.0025 (5)	0.0058 (5)
C4	0.0182 (6)	0.0236 (6)	0.0218 (6)	−0.0034 (5)	0.0035 (5)	−0.0057 (5)
C5	0.0143 (5)	0.0166 (5)	0.0149 (5)	−0.0001 (4)	0.0021 (4)	−0.0010 (4)
C6	0.0171 (6)	0.0228 (6)	0.0201 (6)	0.0007 (5)	0.0058 (4)	−0.0024 (5)
C7	0.0141 (5)	0.0162 (5)	0.0164 (5)	−0.0005 (4)	0.0022 (4)	−0.0012 (4)
C8	0.0183 (6)	0.0219 (6)	0.0170 (5)	−0.0008 (5)	0.0004 (4)	0.0013 (4)
C9	0.0177 (6)	0.0268 (6)	0.0224 (6)	−0.0026 (5)	−0.0024 (5)	−0.0029 (5)
C10	0.0180 (6)	0.0222 (6)	0.0299 (7)	−0.0052 (5)	0.0019 (5)	−0.0020 (5)
C11	0.0241 (6)	0.0236 (6)	0.0261 (6)	−0.0073 (5)	0.0023 (5)	0.0059 (5)
C12	0.0185 (6)	0.0235 (6)	0.0191 (6)	−0.0043 (5)	−0.0014 (4)	0.0039 (5)
C13	0.0239 (6)	0.0193 (6)	0.0314 (7)	0.0015 (5)	0.0029 (5)	−0.0068 (5)
C14	0.0174 (6)	0.0176 (5)	0.0191 (6)	−0.0018 (4)	0.0036 (4)	−0.0029 (4)
C15	0.0211 (6)	0.0277 (6)	0.0207 (6)	−0.0034 (5)	0.0012 (5)	−0.0078 (5)
C16	0.0177 (6)	0.0272 (6)	0.0195 (6)	0.0050 (5)	−0.0006 (4)	−0.0007 (5)
C17	0.0160 (5)	0.0188 (5)	0.0139 (5)	0.0014 (4)	0.0010 (4)	0.0001 (4)
C18	0.0238 (6)	0.0313 (7)	0.0206 (6)	0.0021 (5)	0.0034 (5)	0.0101 (5)
C19	0.0133 (5)	0.0198 (5)	0.0161 (5)	0.0010 (4)	0.0025 (4)	0.0029 (4)
C20	0.0251 (7)	0.0361 (7)	0.0184 (6)	−0.0095 (6)	0.0057 (5)	−0.0044 (5)
C21	0.0296 (7)	0.0532 (9)	0.0182 (6)	−0.0108 (7)	0.0105 (5)	−0.0001 (6)
C22	0.0185 (6)	0.0358 (7)	0.0289 (7)	−0.0031 (5)	0.0071 (5)	0.0111 (6)
C23	0.0194 (6)	0.0198 (6)	0.0340 (7)	−0.0022 (5)	0.0068 (5)	0.0022 (5)
C24	0.0191 (6)	0.0206 (6)	0.0236 (6)	−0.0006 (5)	0.0085 (5)	−0.0011 (5)
Si3	0.01442 (15)	0.01542 (14)	0.01257 (14)	−0.00162 (11)	0.00185 (11)	0.00045 (11)
Si4	0.01289 (15)	0.01534 (14)	0.01194 (14)	−0.00043 (11)	0.00134 (11)	−0.00046 (11)
C25	0.0242 (6)	0.0246 (6)	0.0165 (5)	−0.0027 (5)	0.0028 (5)	−0.0042 (5)
C26	0.0208 (6)	0.0171 (5)	0.0170 (5)	−0.0013 (4)	0.0004 (4)	−0.0015 (4)
C27	0.0284 (7)	0.0268 (6)	0.0249 (6)	−0.0098 (5)	−0.0001 (5)	−0.0032 (5)
C28	0.0286 (7)	0.0293 (7)	0.0316 (7)	−0.0009 (6)	0.0028 (6)	0.0148 (6)
C29	0.0234 (6)	0.0235 (6)	0.0166 (5)	−0.0049 (5)	0.0027 (5)	0.0040 (5)
C30	0.0294 (7)	0.0304 (7)	0.0330 (7)	−0.0038 (6)	0.0135 (6)	0.0083 (6)
C31	0.0129 (5)	0.0178 (5)	0.0189 (5)	−0.0025 (4)	0.0032 (4)	0.0005 (4)
C32	0.0183 (6)	0.0237 (6)	0.0198 (6)	−0.0003 (5)	0.0032 (4)	−0.0002 (5)
C33	0.0223 (6)	0.0244 (6)	0.0262 (6)	0.0006 (5)	0.0089 (5)	−0.0038 (5)
C34	0.0182 (6)	0.0206 (6)	0.0333 (7)	0.0017 (5)	0.0077 (5)	0.0039 (5)
C35	0.0201 (6)	0.0237 (6)	0.0239 (6)	0.0007 (5)	0.0030 (5)	0.0063 (5)
C36	0.0177 (6)	0.0207 (6)	0.0191 (6)	−0.0013 (4)	0.0042 (4)	0.0010 (5)
C37	0.0232 (6)	0.0218 (6)	0.0310 (7)	0.0054 (5)	0.0048 (5)	−0.0021 (5)
C38	0.0146 (5)	0.0203 (5)	0.0181 (5)	0.0019 (4)	0.0012 (4)	0.0039 (4)
C39	0.0152 (6)	0.0281 (6)	0.0269 (6)	0.0013 (5)	0.0002 (5)	0.0053 (5)
C40	0.0292 (7)	0.0248 (6)	0.0271 (7)	−0.0044 (5)	−0.0025 (5)	−0.0079 (5)
C41	0.0174 (6)	0.0263 (6)	0.0166 (5)	−0.0001 (5)	0.0003 (4)	−0.0060 (5)
C42	0.0300 (7)	0.0453 (8)	0.0139 (6)	−0.0067 (6)	0.0018 (5)	−0.0051 (6)
C43	0.0151 (5)	0.0167 (5)	0.0143 (5)	−0.0035 (4)	−0.0001 (4)	0.0002 (4)
C44	0.0176 (6)	0.0253 (6)	0.0176 (6)	0.0000 (5)	0.0022 (4)	0.0010 (5)
C45	0.0235 (6)	0.0342 (7)	0.0172 (6)	−0.0041 (5)	0.0061 (5)	0.0024 (5)
C46	0.0277 (7)	0.0286 (7)	0.0190 (6)	−0.0081 (5)	−0.0001 (5)	0.0067 (5)
C47	0.0255 (7)	0.0194 (6)	0.0253 (6)	−0.0008 (5)	−0.0013 (5)	0.0051 (5)
C48	0.0196 (6)	0.0179 (5)	0.0207 (6)	−0.0016 (4)	0.0027 (4)	0.0005 (4)

### (1) 1,1,2,2-Tetraisopropyl-1,2-diphenyldisilane Geometric parameters (Å, º)

**Table:**

Si1—C7	1.8926 (12)	Si3—C31	1.8911 (12)
Si1—C2	1.9005 (12)	Si3—C26	1.8993 (12)
Si1—C5	1.9107 (12)	Si3—C29	1.9101 (12)
Si1—Si2	2.3898 (4)	Si3—Si4	2.3799 (5)
Si2—C19	1.8926 (12)	Si4—C43	1.8906 (12)
Si2—C14	1.8990 (12)	Si4—C38	1.9045 (12)
Si2—C17	1.9051 (12)	Si4—C41	1.9087 (12)
C1—C2	1.5375 (17)	C25—C26	1.5387 (17)
C1—H1A	0.9800	C25—H25A	0.9800
C1—H1B	0.9800	C25—H25B	0.9800
C1—H1C	0.9800	C25—H25C	0.9800
C2—C3	1.5388 (17)	C26—C27	1.5312 (17)
C2—H2	1.0000	C26—H26	1.0000
C3—H3A	0.9800	C27—H27A	0.9800
C3—H3B	0.9800	C27—H27B	0.9800
C3—H3C	0.9800	C27—H27C	0.9800
C4—C5	1.5367 (16)	C28—C29	1.5340 (19)
C4—H4A	0.9800	C28—H28A	0.9800
C4—H4B	0.9800	C28—H28B	0.9800
C4—H4C	0.9800	C28—H28C	0.9800
C5—C6	1.5347 (16)	C29—C30	1.5379 (18)
C5—H5	1.0000	C29—H29	1.0000
C6—H6A	0.9800	C30—H30A	0.9800
C6—H6B	0.9800	C30—H30B	0.9800
C6—H6C	0.9800	C30—H30C	0.9800
C7—C8	1.3987 (16)	C31—C36	1.4008 (16)
C7—C12	1.4033 (16)	C31—C32	1.4096 (17)
C8—C9	1.3927 (17)	C32—C33	1.3893 (17)
C8—H8	0.9500	C32—H32	0.9500
C9—C10	1.3839 (18)	C33—C34	1.3881 (19)
C9—H9	0.9500	C33—H33	0.9500
C10—C11	1.3864 (18)	C34—C35	1.3860 (19)
C10—H10	0.9500	C34—H34	0.9500
C11—C12	1.3903 (17)	C35—C36	1.3935 (17)
C11—H11	0.9500	C35—H35	0.9500
C12—H12	0.9500	C36—H36	0.9500
C13—C14	1.5357 (17)	C37—C38	1.5350 (17)
C13—H13A	0.9800	C37—H37A	0.9800
C13—H13B	0.9800	C37—H37B	0.9800
C13—H13C	0.9800	C37—H37C	0.9800
C14—C15	1.5342 (17)	C38—C39	1.5375 (17)
C14—H14	1.0000	C38—H38	1.0000
C15—H15A	0.9800	C39—H39A	0.9800
C15—H15B	0.9800	C39—H39B	0.9800
C15—H15C	0.9800	C39—H39C	0.9800
C16—C17	1.5381 (16)	C40—C41	1.5354 (18)
C16—H16A	0.9800	C40—H40A	0.9800
C16—H16B	0.9800	C40—H40B	0.9800
C16—H16C	0.9800	C40—H40C	0.9800
C17—C18	1.5346 (17)	C41—C42	1.5388 (18)
C17—H17	1.0000	C41—H41	1.0000
C18—H18A	0.9800	C42—H42A	0.9800
C18—H18B	0.9800	C42—H42B	0.9800
C18—H18C	0.9800	C42—H42C	0.9800
C19—C20	1.3968 (17)	C43—C44	1.4011 (16)
C19—C24	1.4014 (17)	C43—C48	1.4033 (16)
C20—C21	1.3944 (18)	C44—C45	1.3921 (17)
C20—H20	0.9500	C44—H44	0.9500
C21—C22	1.381 (2)	C45—C46	1.3842 (19)
C21—H21	0.9500	C45—H45	0.9500
C22—C23	1.3801 (19)	C46—C47	1.3851 (19)
C22—H22	0.9500	C46—H46	0.9500
C23—C24	1.3915 (17)	C47—C48	1.3903 (17)
C23—H23	0.9500	C47—H47	0.9500
C24—H24	0.9500	C48—H48	0.9500
			
C7—Si1—C2	112.02 (5)	C31—Si3—C26	112.86 (5)
C7—Si1—C5	107.32 (5)	C31—Si3—C29	106.67 (5)
C2—Si1—C5	109.35 (5)	C26—Si3—C29	109.63 (6)
C7—Si1—Si2	109.25 (4)	C31—Si3—Si4	106.81 (4)
C2—Si1—Si2	110.49 (4)	C26—Si3—Si4	110.18 (4)
C5—Si1—Si2	108.29 (4)	C29—Si3—Si4	110.61 (4)
C19—Si2—C14	112.39 (5)	C43—Si4—C38	112.83 (5)
C19—Si2—C17	106.02 (5)	C43—Si4—C41	106.70 (5)
C14—Si2—C17	110.72 (5)	C38—Si4—C41	109.98 (5)
C19—Si2—Si1	110.02 (4)	C43—Si4—Si3	106.18 (4)
C14—Si2—Si1	108.90 (4)	C38—Si4—Si3	112.11 (4)
C17—Si2—Si1	108.71 (4)	C41—Si4—Si3	108.79 (4)
C2—C1—H1A	109.5	C26—C25—H25A	109.5
C2—C1—H1B	109.5	C26—C25—H25B	109.5
H1A—C1—H1B	109.5	H25A—C25—H25B	109.5
C2—C1—H1C	109.5	C26—C25—H25C	109.5
H1A—C1—H1C	109.5	H25A—C25—H25C	109.5
H1B—C1—H1C	109.5	H25B—C25—H25C	109.5
C1—C2—C3	110.59 (10)	C27—C26—C25	110.58 (10)
C1—C2—Si1	114.42 (8)	C27—C26—Si3	113.72 (9)
C3—C2—Si1	114.16 (8)	C25—C26—Si3	115.18 (8)
C1—C2—H2	105.6	C27—C26—H26	105.5
C3—C2—H2	105.6	C25—C26—H26	105.5
Si1—C2—H2	105.6	Si3—C26—H26	105.5
C2—C3—H3A	109.5	C26—C27—H27A	109.5
C2—C3—H3B	109.5	C26—C27—H27B	109.5
H3A—C3—H3B	109.5	H27A—C27—H27B	109.5
C2—C3—H3C	109.5	C26—C27—H27C	109.5
H3A—C3—H3C	109.5	H27A—C27—H27C	109.5
H3B—C3—H3C	109.5	H27B—C27—H27C	109.5
C5—C4—H4A	109.5	C29—C28—H28A	109.5
C5—C4—H4B	109.5	C29—C28—H28B	109.5
H4A—C4—H4B	109.5	H28A—C28—H28B	109.5
C5—C4—H4C	109.5	C29—C28—H28C	109.5
H4A—C4—H4C	109.5	H28A—C28—H28C	109.5
H4B—C4—H4C	109.5	H28B—C28—H28C	109.5
C6—C5—C4	109.95 (10)	C28—C29—C30	110.32 (11)
C6—C5—Si1	114.32 (8)	C28—C29—Si3	111.38 (9)
C4—C5—Si1	111.81 (8)	C30—C29—Si3	115.26 (9)
C6—C5—H5	106.8	C28—C29—H29	106.4
C4—C5—H5	106.8	C30—C29—H29	106.4
Si1—C5—H5	106.8	Si3—C29—H29	106.4
C5—C6—H6A	109.5	C29—C30—H30A	109.5
C5—C6—H6B	109.5	C29—C30—H30B	109.5
H6A—C6—H6B	109.5	H30A—C30—H30B	109.5
C5—C6—H6C	109.5	C29—C30—H30C	109.5
H6A—C6—H6C	109.5	H30A—C30—H30C	109.5
H6B—C6—H6C	109.5	H30B—C30—H30C	109.5
C8—C7—C12	116.81 (11)	C36—C31—C32	116.65 (11)
C8—C7—Si1	123.61 (9)	C36—C31—Si3	124.02 (9)
C12—C7—Si1	119.57 (9)	C32—C31—Si3	119.33 (9)
C9—C8—C7	121.63 (11)	C33—C32—C31	121.74 (12)
C9—C8—H8	119.2	C33—C32—H32	119.1
C7—C8—H8	119.2	C31—C32—H32	119.1
C10—C9—C8	120.20 (11)	C34—C33—C32	120.27 (12)
C10—C9—H9	119.9	C34—C33—H33	119.9
C8—C9—H9	119.9	C32—C33—H33	119.9
C9—C10—C11	119.59 (11)	C35—C34—C33	119.26 (12)
C9—C10—H10	120.2	C35—C34—H34	120.4
C11—C10—H10	120.2	C33—C34—H34	120.4
C10—C11—C12	119.90 (12)	C34—C35—C36	120.41 (12)
C10—C11—H11	120.0	C34—C35—H35	119.8
C12—C11—H11	120.0	C36—C35—H35	119.8
C11—C12—C7	121.87 (11)	C35—C36—C31	121.67 (11)
C11—C12—H12	119.1	C35—C36—H36	119.2
C7—C12—H12	119.1	C31—C36—H36	119.2
C14—C13—H13A	109.5	C38—C37—H37A	109.5
C14—C13—H13B	109.5	C38—C37—H37B	109.5
H13A—C13—H13B	109.5	H37A—C37—H37B	109.5
C14—C13—H13C	109.5	C38—C37—H37C	109.5
H13A—C13—H13C	109.5	H37A—C37—H37C	109.5
H13B—C13—H13C	109.5	H37B—C37—H37C	109.5
C15—C14—C13	110.61 (10)	C37—C38—C39	109.36 (10)
C15—C14—Si2	113.92 (8)	C37—C38—Si4	115.80 (8)
C13—C14—Si2	114.15 (8)	C39—C38—Si4	114.52 (8)
C15—C14—H14	105.8	C37—C38—H38	105.4
C13—C14—H14	105.8	C39—C38—H38	105.4
Si2—C14—H14	105.8	Si4—C38—H38	105.4
C14—C15—H15A	109.5	C38—C39—H39A	109.5
C14—C15—H15B	109.5	C38—C39—H39B	109.5
H15A—C15—H15B	109.5	H39A—C39—H39B	109.5
C14—C15—H15C	109.5	C38—C39—H39C	109.5
H15A—C15—H15C	109.5	H39A—C39—H39C	109.5
H15B—C15—H15C	109.5	H39B—C39—H39C	109.5
C17—C16—H16A	109.5	C41—C40—H40A	109.5
C17—C16—H16B	109.5	C41—C40—H40B	109.5
H16A—C16—H16B	109.5	H40A—C40—H40B	109.5
C17—C16—H16C	109.5	C41—C40—H40C	109.5
H16A—C16—H16C	109.5	H40A—C40—H40C	109.5
H16B—C16—H16C	109.5	H40B—C40—H40C	109.5
C18—C17—C16	109.76 (10)	C40—C41—C42	109.78 (10)
C18—C17—Si2	112.38 (8)	C40—C41—Si4	114.00 (9)
C16—C17—Si2	114.43 (8)	C42—C41—Si4	112.92 (9)
C18—C17—H17	106.6	C40—C41—H41	106.5
C16—C17—H17	106.6	C42—C41—H41	106.5
Si2—C17—H17	106.6	Si4—C41—H41	106.5
C17—C18—H18A	109.5	C41—C42—H42A	109.5
C17—C18—H18B	109.5	C41—C42—H42B	109.5
H18A—C18—H18B	109.5	H42A—C42—H42B	109.5
C17—C18—H18C	109.5	C41—C42—H42C	109.5
H18A—C18—H18C	109.5	H42A—C42—H42C	109.5
H18B—C18—H18C	109.5	H42B—C42—H42C	109.5
C20—C19—C24	116.76 (11)	C44—C43—C48	116.82 (11)
C20—C19—Si2	124.48 (9)	C44—C43—Si4	123.77 (9)
C24—C19—Si2	118.76 (9)	C48—C43—Si4	119.38 (9)
C21—C20—C19	121.49 (13)	C45—C44—C43	121.81 (12)
C21—C20—H20	119.3	C45—C44—H44	119.1
C19—C20—H20	119.3	C43—C44—H44	119.1
C22—C21—C20	120.16 (13)	C46—C45—C44	119.92 (12)
C22—C21—H21	119.9	C46—C45—H45	120.0
C20—C21—H21	119.9	C44—C45—H45	120.0
C23—C22—C21	119.85 (12)	C45—C46—C47	119.71 (12)
C23—C22—H22	120.1	C45—C46—H46	120.1
C21—C22—H22	120.1	C47—C46—H46	120.1
C22—C23—C24	119.69 (12)	C46—C47—C48	120.13 (12)
C22—C23—H23	120.2	C46—C47—H47	119.9
C24—C23—H23	120.2	C48—C47—H47	119.9
C23—C24—C19	122.00 (12)	C47—C48—C43	121.61 (12)
C23—C24—H24	119.0	C47—C48—H48	119.2
C19—C24—H24	119.0	C43—C48—H48	119.2
			
C2—Si1—C7—C8	−13.73 (12)	C31—Si3—C26—C27	55.71 (10)
C5—Si1—C7—C8	−133.76 (10)	C29—Si3—C26—C27	−63.04 (10)
Si2—Si1—C7—C8	109.03 (10)	Si4—Si3—C26—C27	175.01 (8)
C2—Si1—C7—C12	166.95 (9)	C31—Si3—C26—C25	−73.40 (10)
C5—Si1—C7—C12	46.92 (11)	C29—Si3—C26—C25	167.85 (9)
Si2—Si1—C7—C12	−70.28 (10)	Si4—Si3—C26—C25	45.90 (10)
C12—C7—C8—C9	0.23 (18)	C26—Si3—C31—C36	18.23 (12)
Si1—C7—C8—C9	−179.10 (10)	C29—Si3—C31—C36	138.69 (10)
C7—C8—C9—C10	−0.42 (19)	Si4—Si3—C31—C36	−103.00 (10)
C8—C9—C10—C11	0.2 (2)	C26—Si3—C31—C32	−161.78 (9)
C9—C10—C11—C12	0.2 (2)	C29—Si3—C31—C32	−41.32 (11)
C10—C11—C12—C7	−0.4 (2)	Si4—Si3—C31—C32	76.99 (9)
C8—C7—C12—C11	0.17 (18)	C36—C31—C32—C33	−0.53 (18)
Si1—C7—C12—C11	179.53 (10)	Si3—C31—C32—C33	179.48 (10)
C19—Si2—C14—C15	66.80 (10)	C31—C32—C33—C34	−0.02 (19)
C17—Si2—C14—C15	−174.84 (8)	C32—C33—C34—C35	0.44 (19)
Si1—Si2—C14—C15	−55.37 (9)	C33—C34—C35—C36	−0.29 (19)
C19—Si2—C14—C13	−61.59 (10)	C34—C35—C36—C31	−0.29 (19)
C17—Si2—C14—C13	56.77 (10)	C32—C31—C36—C35	0.69 (17)
Si1—Si2—C14—C13	176.24 (8)	Si3—C31—C36—C35	−179.32 (9)
C14—Si2—C19—C20	−11.15 (13)	C38—Si4—C43—C44	−6.11 (12)
C17—Si2—C19—C20	−132.24 (11)	C41—Si4—C43—C44	−127.00 (10)
Si1—Si2—C19—C20	110.39 (11)	Si3—Si4—C43—C44	117.08 (10)
C14—Si2—C19—C24	169.45 (9)	C38—Si4—C43—C48	175.65 (9)
C17—Si2—C19—C24	48.35 (11)	C41—Si4—C43—C48	54.76 (10)
Si1—Si2—C19—C24	−69.02 (10)	Si3—Si4—C43—C48	−61.16 (10)
C24—C19—C20—C21	2.1 (2)	C48—C43—C44—C45	−0.07 (18)
Si2—C19—C20—C21	−177.28 (11)	Si4—C43—C44—C45	−178.34 (10)
C19—C20—C21—C22	−1.0 (2)	C43—C44—C45—C46	0.94 (19)
C20—C21—C22—C23	−1.1 (2)	C44—C45—C46—C47	−1.0 (2)
C21—C22—C23—C24	2.0 (2)	C45—C46—C47—C48	0.2 (2)
C22—C23—C24—C19	−0.8 (2)	C46—C47—C48—C43	0.70 (19)
C20—C19—C24—C23	−1.23 (18)	C44—C43—C48—C47	−0.75 (17)
Si2—C19—C24—C23	178.22 (10)	Si4—C43—C48—C47	177.61 (9)

### (2) 1,1,2,2-Tetrabromoisopropyl-1,2-diphenyldisilane Crystal data

**Table:**

C_24_H_34_Br_4_Si_2_	*F*(000) = 1384
*M**_r_* = 698.33	*D*_x_ = 1.691 Mg m^−^^3^
Monoclinic, *P*2_1_/*c*	Mo *K*α radiation, λ = 0.71073 Å
*a* = 8.8779 (7) Å	Cell parameters from 9033 reflections
*b* = 10.4042 (8) Å	θ = 2.4–28.4°
*c* = 29.699 (2) Å	µ = 5.97 mm^−^^1^
β = 90.975 (1)°	*T* = 100 K
*V* = 2742.8 (4) Å^3^	Thick plate, colorless
*Z* = 4	0.14 × 0.12 × 0.07 mm

### (2) 1,1,2,2-Tetrabromoisopropyl-1,2-diphenyldisilane Data collection

**Table:**

Bruker SMART APEX CCD diffractometer	6875 independent reflections
Radiation source: fine-focus sealed tube	5471 reflections with *I* > 2σ(*I*)
Graphite monochromator	*R*_int_ = 0.052
φ and ω scans	θ_max_ = 28.4°, θ_min_ = 2.1°
Absorption correction: multi-scan (*SADABS*; Bruker, 2016)	*h* = −11→11
*T*_min_ = 0.49, *T*_max_ = 0.69	*k* = −13→13
47392 measured reflections	*l* = −39→39

### (2) 1,1,2,2-Tetrabromoisopropyl-1,2-diphenyldisilane Refinement

**Table:**

Refinement on *F*^2^	Primary atom site location: structure-invariant direct methods
Least-squares matrix: full	Secondary atom site location: difference Fourier map
*R*[*F*^2^ > 2σ(*F*^2^)] = 0.028	Hydrogen site location: inferred from neighbouring sites
*wR*(*F*^2^) = 0.062	H-atom parameters constrained
*S* = 1.03	*w* = 1/[σ^2^(*F*_o_^2^) + (0.0222*P*)^2^ + 2.5797*P*] where *P* = (*F*_o_^2^ + 2*F*_c_^2^)/3
6875 reflections	(Δ/σ)_max_ = 0.001
287 parameters	Δρ_max_ = 1.00 e Å^−^^3^
38 restraints	Δρ_min_ = −1.05 e Å^−^^3^

### (2) 1,1,2,2-Tetrabromoisopropyl-1,2-diphenyldisilane Special details

**Table:**

Experimental. The diffraction data were obtained from 3 sets of 400 frames, each of width 0.5° in ω, colllected at φ = 0.00, 90.00 and 180.00° and 2 sets of 800 frames, each of width 0.45° in φ, collected at ω = –30.00 and 210.00°. The scan time was 20 sec/frame.
Geometry. All esds (except the esd in the dihedral angle between two l.s. planes) are estimated using the full covariance matrix. The cell esds are taken into account individually in the estimation of esds in distances, angles and torsion angles; correlations between esds in cell parameters are only used when they are defined by crystal symmetry. An approximate (isotropic) treatment of cell esds is used for estimating esds involving l.s. planes.
Refinement. Refinement of F^2^ against ALL reflections. The weighted R-factor wR and goodness of fit S are based on F^2^, conventional R-factors R are based on F, with F set to zero for negative F^2^. The threshold expression of F^2^ > 2sigma(F^2^) is used only for calculating R-factors(gt) etc. and is not relevant to the choice of reflections for refinement. R-factors based on F^2^ are statistically about twice as large as those based on F, and R- factors based on ALL data will be even larger. H-atoms attached to carbon were placed in calculated positions (C—H = 0.95 - 0.98 Å). All were included as riding contributions with isotropic displacement parameters 1.2 - 1.5 times those of the attached atoms. The bromodimethyl group based on C16 is rotationally disordered over two nearly superimposable sites in an 88:12 ratio. The two components of the disorder were refined subject to restraints that their geometries be comparable.

### (2) 1,1,2,2-Tetrabromoisopropyl-1,2-diphenyldisilane Fractional atomic coordinates and isotropic or equivalent isotropic displacement parameters (Å^2^)

**Table:**

	*x*	*y*	*z*	*U*_iso_*/*U*_eq_	Occ. (<1)
Br1	0.02503 (3)	−0.16520 (3)	0.33755 (2)	0.02338 (7)	
Br2	0.50578 (3)	0.15264 (3)	0.28016 (2)	0.02263 (7)	
Br3	0.01829 (3)	0.41338 (3)	0.33412 (2)	0.02135 (7)	
Si1	0.23670 (7)	0.08194 (7)	0.34048 (2)	0.01256 (14)	
Si2	0.28642 (7)	0.27865 (7)	0.38516 (2)	0.01214 (13)	
C1	0.0249 (3)	0.0278 (2)	0.34221 (8)	0.0167 (5)	
C2	−0.0765 (3)	0.0699 (3)	0.30209 (9)	0.0227 (6)	
H2A	−0.1798	0.0402	0.3069	0.034*	
H2B	−0.0384	0.0325	0.2742	0.034*	
H2C	−0.0758	0.1639	0.2997	0.034*	
C3	−0.0557 (3)	0.0586 (3)	0.38559 (9)	0.0207 (6)	
H3A	−0.0742	0.1513	0.3873	0.031*	
H3B	0.0071	0.0318	0.4114	0.031*	
H3C	−0.1520	0.0126	0.3861	0.031*	
C4	0.2913 (3)	0.0898 (3)	0.27793 (8)	0.0171 (5)	
C5	0.2928 (3)	−0.0419 (3)	0.25465 (9)	0.0233 (6)	
H5A	0.1897	−0.0752	0.2522	0.035*	
H5B	0.3551	−0.1017	0.2724	0.035*	
H5C	0.3345	−0.0329	0.2245	0.035*	
C6	0.2083 (3)	0.1864 (3)	0.24738 (9)	0.0243 (6)	
H6A	0.2686	0.2034	0.2207	0.037*	
H6B	0.1928	0.2667	0.2639	0.037*	
H6C	0.1105	0.1507	0.2380	0.037*	
C7	0.3517 (3)	−0.0469 (2)	0.37044 (8)	0.0144 (5)	
C8	0.4842 (3)	−0.1028 (2)	0.35435 (9)	0.0180 (5)	
H8	0.5176	−0.0806	0.3251	0.022*	
C9	0.5678 (3)	−0.1897 (3)	0.38002 (9)	0.0215 (6)	
H9	0.6568	−0.2261	0.3681	0.026*	
C10	0.5231 (3)	−0.2238 (3)	0.42260 (9)	0.0219 (6)	
H10	0.5815	−0.2822	0.4402	0.026*	
C11	0.3912 (3)	−0.1715 (3)	0.43940 (9)	0.0210 (6)	
H11	0.3587	−0.1943	0.4687	0.025*	
C12	0.3071 (3)	−0.0861 (2)	0.41333 (8)	0.0174 (5)	
H12	0.2160	−0.0529	0.4250	0.021*	
C13	0.2337 (3)	0.4393 (2)	0.35601 (9)	0.0180 (5)	
C14	0.3172 (3)	0.4782 (3)	0.31345 (9)	0.0249 (6)	
H14A	0.2579	0.5425	0.2968	0.037*	
H14B	0.3317	0.4024	0.2944	0.037*	
H14C	0.4155	0.5146	0.3219	0.037*	
C15	0.2331 (3)	0.5527 (3)	0.38851 (10)	0.0259 (6)	
H15A	0.3356	0.5675	0.4002	0.039*	
H15B	0.1663	0.5336	0.4136	0.039*	
H15C	0.1972	0.6297	0.3727	0.039*	
C16	0.4955 (3)	0.2899 (2)	0.40556 (7)	0.0183 (5)	
Br4	0.50066 (3)	0.39238 (3)	0.46210 (2)	0.01971 (9)	0.8812 (9)
C17	0.5787 (3)	0.1649 (2)	0.41677 (13)	0.0185 (6)	0.8812 (9)
H17A	0.5162	0.1116	0.4362	0.028*	0.8812 (9)
H17B	0.5993	0.1183	0.3889	0.028*	0.8812 (9)
H17C	0.6740	0.1849	0.4324	0.028*	0.8812 (9)
C18	0.6092 (5)	0.3659 (3)	0.37416 (12)	0.0237 (10)	0.8812 (9)
H18A	0.5644	0.4484	0.3653	0.036*	0.8812 (9)
H18B	0.7039	0.3814	0.3907	0.036*	0.8812 (9)
H18C	0.6293	0.3148	0.3472	0.036*	0.8812 (9)
Br4A	0.6209 (3)	0.4004 (3)	0.37171 (11)	0.01971 (9)	0.1188 (9)
C17A	0.464 (2)	0.3531 (13)	0.4519 (3)	0.0185 (6)	0.1188 (9)
H17D	0.4135	0.4358	0.4472	0.028*	0.1188 (9)
H17E	0.4000	0.2964	0.4696	0.028*	0.1188 (9)
H17F	0.5599	0.3671	0.4682	0.028*	0.1188 (9)
C18A	0.575 (2)	0.1610 (11)	0.4144 (8)	0.0237 (10)	0.1188 (9)
H18D	0.5960	0.1190	0.3856	0.036*	0.1188 (9)
H18E	0.6699	0.1761	0.4309	0.036*	0.1188 (9)
H18F	0.5099	0.1055	0.4323	0.036*	0.1188 (9)
C19	0.1718 (3)	0.2614 (2)	0.43854 (8)	0.0148 (5)	
C20	0.0389 (3)	0.3299 (3)	0.44706 (9)	0.0208 (6)	
H20	−0.0002	0.3869	0.4248	0.025*	
C21	−0.0368 (3)	0.3163 (3)	0.48729 (9)	0.0257 (6)	
H21	−0.1256	0.3648	0.4923	0.031*	
C22	0.0156 (3)	0.2331 (3)	0.52014 (9)	0.0272 (6)	
H22	−0.0364	0.2243	0.5477	0.033*	
C23	0.1445 (3)	0.1626 (3)	0.51256 (9)	0.0249 (6)	
H23	0.1805	0.1037	0.5347	0.030*	
C24	0.2215 (3)	0.1777 (3)	0.47253 (8)	0.0182 (5)	
H24	0.3109	0.1295	0.4681	0.022*	

### (2) 1,1,2,2-Tetrabromoisopropyl-1,2-diphenyldisilane Atomic displacement parameters (Å^2^)

**Table:**

	*U*^11^	*U*^22^	*U*^33^	*U*^12^	*U*^13^	*U*^23^
Br1	0.02017 (13)	0.01707 (13)	0.03266 (15)	−0.00137 (10)	−0.00704 (11)	−0.00487 (11)
Br2	0.01913 (13)	0.02930 (16)	0.01961 (13)	0.00066 (11)	0.00462 (10)	0.00286 (11)
Br3	0.01822 (13)	0.02552 (15)	0.02019 (13)	0.00314 (10)	−0.00308 (10)	0.00377 (11)
Si1	0.0119 (3)	0.0145 (3)	0.0112 (3)	0.0016 (3)	−0.0008 (2)	−0.0004 (3)
Si2	0.0125 (3)	0.0135 (3)	0.0105 (3)	0.0000 (3)	0.0009 (2)	−0.0001 (3)
C1	0.0160 (12)	0.0139 (12)	0.0200 (13)	0.0010 (10)	−0.0032 (10)	−0.0017 (10)
C2	0.0164 (13)	0.0244 (15)	0.0271 (14)	0.0039 (11)	−0.0062 (11)	−0.0013 (12)
C3	0.0141 (13)	0.0220 (14)	0.0260 (14)	−0.0027 (10)	0.0034 (10)	−0.0012 (11)
C4	0.0168 (12)	0.0235 (14)	0.0110 (11)	0.0032 (10)	0.0003 (9)	−0.0014 (10)
C5	0.0259 (14)	0.0292 (16)	0.0148 (13)	0.0045 (12)	0.0001 (11)	−0.0079 (11)
C6	0.0285 (15)	0.0315 (16)	0.0129 (12)	0.0057 (12)	−0.0013 (11)	0.0030 (11)
C7	0.0146 (12)	0.0121 (12)	0.0165 (12)	−0.0011 (9)	−0.0027 (9)	−0.0021 (10)
C8	0.0187 (13)	0.0180 (13)	0.0172 (12)	0.0001 (10)	−0.0026 (10)	−0.0004 (10)
C9	0.0160 (13)	0.0187 (14)	0.0297 (15)	0.0028 (10)	−0.0038 (11)	−0.0021 (11)
C10	0.0218 (14)	0.0159 (13)	0.0275 (14)	0.0003 (11)	−0.0109 (11)	0.0026 (11)
C11	0.0266 (14)	0.0181 (13)	0.0183 (13)	−0.0035 (11)	−0.0050 (11)	0.0059 (11)
C12	0.0173 (12)	0.0153 (13)	0.0195 (13)	−0.0030 (10)	−0.0019 (10)	−0.0015 (10)
C13	0.0152 (12)	0.0178 (13)	0.0209 (13)	0.0008 (10)	−0.0035 (10)	0.0017 (11)
C14	0.0257 (15)	0.0263 (15)	0.0226 (14)	−0.0025 (12)	0.0020 (11)	0.0095 (12)
C15	0.0333 (16)	0.0134 (14)	0.0309 (16)	0.0010 (11)	−0.0035 (12)	0.0013 (12)
C16	0.0190 (13)	0.0205 (14)	0.0154 (12)	−0.0018 (10)	−0.0007 (10)	−0.0055 (10)
Br4	0.02271 (16)	0.02007 (17)	0.01624 (15)	−0.00242 (12)	−0.00282 (11)	−0.00367 (12)
C17	0.0059 (12)	0.0196 (15)	0.0300 (16)	0.0003 (11)	−0.0014 (11)	−0.0114 (13)
C18	0.041 (2)	0.0069 (19)	0.0240 (18)	0.0023 (15)	0.0040 (14)	0.0091 (15)
Br4A	0.02271 (16)	0.02007 (17)	0.01624 (15)	−0.00242 (12)	−0.00282 (11)	−0.00367 (12)
C17A	0.0059 (12)	0.0196 (15)	0.0300 (16)	0.0003 (11)	−0.0014 (11)	−0.0114 (13)
C18A	0.041 (2)	0.0069 (19)	0.0240 (18)	0.0023 (15)	0.0040 (14)	0.0091 (15)
C19	0.0163 (12)	0.0146 (12)	0.0135 (12)	−0.0020 (10)	−0.0003 (9)	−0.0034 (10)
C20	0.0204 (13)	0.0261 (15)	0.0159 (12)	−0.0007 (11)	0.0011 (10)	−0.0015 (11)
C21	0.0195 (14)	0.0328 (17)	0.0251 (15)	0.0005 (12)	0.0077 (11)	−0.0046 (12)
C22	0.0269 (15)	0.0392 (18)	0.0156 (13)	−0.0085 (13)	0.0081 (11)	−0.0025 (12)
C23	0.0263 (15)	0.0319 (16)	0.0166 (13)	−0.0075 (12)	0.0031 (11)	0.0041 (12)
C24	0.0177 (13)	0.0186 (13)	0.0182 (13)	−0.0012 (10)	0.0020 (10)	0.0011 (11)

### (2) 1,1,2,2-Tetrabromoisopropyl-1,2-diphenyldisilane Geometric parameters (Å, º)

**Table:**

Br1—C1	2.013 (3)	C13—C15	1.524 (4)
Br2—C4	2.014 (3)	C13—C14	1.531 (4)
Br3—C13	2.027 (2)	C14—H14A	0.9800
Si1—C7	1.897 (2)	C14—H14B	0.9800
Si1—C4	1.929 (3)	C14—H14C	0.9800
Si1—C1	1.965 (3)	C15—H15A	0.9800
Si1—Si2	2.4746 (10)	C15—H15B	0.9800
Si2—C19	1.907 (3)	C15—H15C	0.9800
Si2—C13	1.936 (3)	C16—C17	1.529 (3)
Si2—C16	1.946 (2)	C16—C18A	1.536 (6)
C1—C3	1.518 (4)	C16—C17A	1.555 (6)
C1—C2	1.544 (3)	C16—C18	1.596 (3)
C2—H2A	0.9800	C16—Br4A	1.901 (3)
C2—H2B	0.9800	C16—Br4	1.989 (2)
C2—H2C	0.9800	C17—H17A	0.9800
C3—H3A	0.9800	C17—H17B	0.9800
C3—H3B	0.9800	C17—H17C	0.9800
C3—H3C	0.9800	C18—H18A	0.9800
C4—C6	1.534 (3)	C18—H18B	0.9800
C4—C5	1.535 (4)	C18—H18C	0.9800
C5—H5A	0.9800	C17A—H17D	0.9800
C5—H5B	0.9800	C17A—H17E	0.9800
C5—H5C	0.9800	C17A—H17F	0.9800
C6—H6A	0.9800	C18A—H18D	0.9800
C6—H6B	0.9800	C18A—H18E	0.9800
C6—H6C	0.9800	C18A—H18F	0.9800
C7—C12	1.401 (3)	C19—C24	1.399 (3)
C7—C8	1.403 (3)	C19—C20	1.405 (4)
C8—C9	1.389 (3)	C20—C21	1.388 (4)
C8—H8	0.9500	C20—H20	0.9500
C9—C10	1.378 (4)	C21—C22	1.380 (4)
C9—H9	0.9500	C21—H21	0.9500
C10—C11	1.391 (4)	C22—C23	1.381 (4)
C10—H10	0.9500	C22—H22	0.9500
C11—C12	1.388 (3)	C23—C24	1.390 (4)
C11—H11	0.9500	C23—H23	0.9500
C12—H12	0.9500	C24—H24	0.9500
			
C7—Si1—C4	109.83 (11)	C15—C13—Br3	107.03 (18)
C7—Si1—C1	107.04 (11)	C14—C13—Br3	103.77 (16)
C4—Si1—C1	107.07 (11)	Si2—C13—Br3	104.37 (12)
C7—Si1—Si2	104.08 (8)	C13—C14—H14A	109.5
C4—Si1—Si2	115.84 (9)	C13—C14—H14B	109.5
C1—Si1—Si2	112.65 (8)	H14A—C14—H14B	109.5
C19—Si2—C13	108.93 (11)	C13—C14—H14C	109.5
C19—Si2—C16	105.45 (10)	H14A—C14—H14C	109.5
C13—Si2—C16	108.03 (10)	H14B—C14—H14C	109.5
C19—Si2—Si1	105.98 (8)	C13—C15—H15A	109.5
C13—Si2—Si1	115.77 (8)	C13—C15—H15B	109.5
C16—Si2—Si1	112.15 (7)	H15A—C15—H15B	109.5
C3—C1—C2	108.5 (2)	C13—C15—H15C	109.5
C3—C1—Si1	115.27 (17)	H15A—C15—H15C	109.5
C2—C1—Si1	116.37 (18)	H15B—C15—H15C	109.5
C3—C1—Br1	105.64 (17)	C18A—C16—C17A	107.8 (6)
C2—C1—Br1	103.36 (16)	C17—C16—C18	103.9 (2)
Si1—C1—Br1	106.40 (12)	C18A—C16—Br4A	110.3 (4)
C1—C2—H2A	109.5	C17A—C16—Br4A	109.1 (4)
C1—C2—H2B	109.5	C17—C16—Si2	118.14 (18)
H2A—C2—H2B	109.5	C18A—C16—Si2	115.7 (10)
C1—C2—H2C	109.5	C17A—C16—Si2	96.8 (8)
H2A—C2—H2C	109.5	C18—C16—Si2	117.2 (2)
H2B—C2—H2C	109.5	Br4A—C16—Si2	115.84 (15)
C1—C3—H3A	109.5	C17—C16—Br4	105.53 (19)
C1—C3—H3B	109.5	C18—C16—Br4	102.80 (18)
H3A—C3—H3B	109.5	Si2—C16—Br4	107.65 (11)
C1—C3—H3C	109.5	C16—C17—H17A	109.5
H3A—C3—H3C	109.5	C16—C17—H17B	109.5
H3B—C3—H3C	109.5	H17A—C17—H17B	109.5
C6—C4—C5	109.0 (2)	C16—C17—H17C	109.5
C6—C4—Si1	118.12 (18)	H17A—C17—H17C	109.5
C5—C4—Si1	113.59 (18)	H17B—C17—H17C	109.5
C6—C4—Br2	104.53 (18)	C16—C18—H18A	109.5
C5—C4—Br2	106.82 (17)	C16—C18—H18B	109.5
Si1—C4—Br2	103.59 (11)	H18A—C18—H18B	109.5
C4—C5—H5A	109.5	C16—C18—H18C	109.5
C4—C5—H5B	109.5	H18A—C18—H18C	109.5
H5A—C5—H5B	109.5	H18B—C18—H18C	109.5
C4—C5—H5C	109.5	C16—C17A—H17D	109.5
H5A—C5—H5C	109.5	C16—C17A—H17E	109.5
H5B—C5—H5C	109.5	H17D—C17A—H17E	109.5
C4—C6—H6A	109.5	C16—C17A—H17F	109.5
C4—C6—H6B	109.5	H17D—C17A—H17F	109.5
H6A—C6—H6B	109.5	H17E—C17A—H17F	109.5
C4—C6—H6C	109.5	C16—C18A—H18D	109.5
H6A—C6—H6C	109.5	C16—C18A—H18E	109.5
H6B—C6—H6C	109.5	H18D—C18A—H18E	109.5
C12—C7—C8	116.1 (2)	C16—C18A—H18F	109.5
C12—C7—Si1	118.27 (19)	H18D—C18A—H18F	109.5
C8—C7—Si1	125.50 (19)	H18E—C18A—H18F	109.5
C9—C8—C7	121.7 (2)	C24—C19—C20	116.2 (2)
C9—C8—H8	119.1	C24—C19—Si2	119.50 (19)
C7—C8—H8	119.1	C20—C19—Si2	124.32 (19)
C10—C9—C8	120.7 (3)	C21—C20—C19	121.6 (3)
C10—C9—H9	119.6	C21—C20—H20	119.2
C8—C9—H9	119.6	C19—C20—H20	119.2
C9—C10—C11	119.1 (2)	C22—C21—C20	120.7 (3)
C9—C10—H10	120.5	C22—C21—H21	119.6
C11—C10—H10	120.5	C20—C21—H21	119.6
C12—C11—C10	119.9 (3)	C21—C22—C23	119.2 (3)
C12—C11—H11	120.1	C21—C22—H22	120.4
C10—C11—H11	120.1	C23—C22—H22	120.4
C11—C12—C7	122.4 (2)	C22—C23—C24	120.0 (3)
C11—C12—H12	118.8	C22—C23—H23	120.0
C7—C12—H12	118.8	C24—C23—H23	120.0
C15—C13—C14	109.0 (2)	C23—C24—C19	122.3 (2)
C15—C13—Si2	112.85 (17)	C23—C24—H24	118.9
C14—C13—Si2	118.71 (19)	C19—C24—H24	118.9
			
C4—Si1—C7—C12	−166.77 (18)	C10—C11—C12—C7	−1.6 (4)
C1—Si1—C7—C12	−50.9 (2)	C8—C7—C12—C11	2.3 (4)
Si2—Si1—C7—C12	68.61 (19)	Si1—C7—C12—C11	−174.4 (2)
C4—Si1—C7—C8	16.8 (2)	C24—C19—C20—C21	1.0 (4)
C1—Si1—C7—C8	132.7 (2)	Si2—C19—C20—C21	−177.8 (2)
Si2—Si1—C7—C8	−107.8 (2)	C19—C20—C21—C22	−0.9 (4)
C12—C7—C8—C9	−1.4 (4)	C20—C21—C22—C23	−0.2 (4)
Si1—C7—C8—C9	175.0 (2)	C21—C22—C23—C24	1.2 (4)
C7—C8—C9—C10	−0.2 (4)	C22—C23—C24—C19	−1.1 (4)
C8—C9—C10—C11	1.1 (4)	C20—C19—C24—C23	0.0 (4)
C9—C10—C11—C12	−0.2 (4)	Si2—C19—C24—C23	178.8 (2)

### (2) 1,1,2,2-Tetrabromoisopropyl-1,2-diphenyldisilane Hydrogen-bond geometry (Å, º)

**Table:**

*D*—H···*A*	*D*—H	H···*A*	*D*···*A*	*D*—H···*A*
C15—H15*C*···Br1^i^	0.98	2.82	3.771 (3)	166

Symmetry code: (i) *x*, *y*+1, *z*.

### (3) 1,2-Di-tert-butyl-1,1,2,2-tetraphenyldisilane Crystal data

**Table:**

C_32_H_38_Si_2_	*F*(000) = 516
*M**_r_* = 478.80	*D*_x_ = 1.182 Mg m^−^^3^
Monoclinic, *P*2_1_/*c*	Mo *K*α radiation, λ = 0.71073 Å
*a* = 8.5622 (5) Å	Cell parameters from 9973 reflections
*b* = 10.2107 (6) Å	θ = 2.4–29.3°
*c* = 15.4586 (10) Å	µ = 0.15 mm^−^^1^
β = 95.452 (1)°	*T* = 100 K
*V* = 1345.37 (14) Å^3^	Block, colorless
*Z* = 2	0.17 × 0.15 × 0.13 mm

### (3) 1,2-Di-tert-butyl-1,1,2,2-tetraphenyldisilane Data collection

**Table:**

Bruker SMART APEX CCD diffractometer	3566 independent reflections
Radiation source: fine-focus sealed tube	3065 reflections with *I* > 2σ(*I*)
Graphite monochromator	*R*_int_ = 0.031
φ and ω scans	θ_max_ = 29.5°, θ_min_ = 2.4°
Absorption correction: multi-scan (*SADABS*; Bruker, 2016)	*h* = −11→11
*T*_min_ = 0.98, *T*_max_ = 0.98	*k* = −13→14
23513 measured reflections	*l* = −21→21

### (3) 1,2-Di-tert-butyl-1,1,2,2-tetraphenyldisilane Refinement

**Table:**

Refinement on *F*^2^	Primary atom site location: structure-invariant direct methods
Least-squares matrix: full	Secondary atom site location: difference Fourier map
*R*[*F*^2^ > 2σ(*F*^2^)] = 0.034	Hydrogen site location: inferred from neighbouring sites
*wR*(*F*^2^) = 0.092	H-atom parameters constrained
*S* = 1.06	*w* = 1/[σ^2^(*F*_o_^2^) + (0.0409*P*)^2^ + 0.5552*P*] where *P* = (*F*_o_^2^ + 2*F*_c_^2^)/3
3566 reflections	(Δ/σ)_max_ = 0.001
179 parameters	Δρ_max_ = 0.38 e Å^−^^3^
43 restraints	Δρ_min_ = −0.29 e Å^−^^3^

### (3) 1,2-Di-tert-butyl-1,1,2,2-tetraphenyldisilane Special details

**Table:**

Experimental. The diffraction data were obtained from 3 sets of 400 frames, each of width 0.5° in ω, colllected at φ = 0.00, 90.00 and 180.00° and 2 sets of 800 frames, each of width 0.45° in φ, collected at ω = –30.00 and 210.00°. The scan time was 20 sec/frame.
Geometry. All esds (except the esd in the dihedral angle between two l.s. planes) are estimated using the full covariance matrix. The cell esds are taken into account individually in the estimation of esds in distances, angles and torsion angles; correlations between esds in cell parameters are only used when they are defined by crystal symmetry. An approximate (isotropic) treatment of cell esds is used for estimating esds involving l.s. planes.
Refinement. Refinement of F^2^ against ALL reflections. The weighted R-factor wR and goodness of fit S are based on F^2^, conventional R-factors R are based on F, with F set to zero for negative F^2^. The threshold expression of F^2^ > 2sigma(F^2^) is used only for calculating R-factors(gt) etc. and is not relevant to the choice of reflections for refinement. R-factors based on F^2^ are statistically about twice as large as those based on F, and R- factors based on ALL data will be even larger. H-atoms attached to carbon were placed in calculated positions (C—H = 0.95 - 0.98 Å). All were included as riding contributions with isotropic displacement parameters 1.2 - 1.5 times those of the attached atoms. The centrosymmetric molecule is disordered over two orientations about the center in a 96:4 ratio. The two components of the disorder were refined subject to restraints that their geometries be comparable. In addition, the phenyl ring of the minor component overlapping with one from the major component was refined as a rigid hexagon.

### (3) 1,2-Di-tert-butyl-1,1,2,2-tetraphenyldisilane Fractional atomic coordinates and isotropic or equivalent isotropic displacement parameters (Å^2^)

**Table:**

	*x*	*y*	*z*	*U*_iso_*/*U*_eq_	Occ. (<1)
Si1	0.97798 (4)	0.05498 (3)	0.06638 (2)	0.01404 (9)	0.9645 (7)
C1	1.17324 (13)	0.10306 (10)	0.12738 (7)	0.0163 (2)	0.9645 (7)
C2	1.24616 (14)	0.03226 (11)	0.19771 (7)	0.0191 (2)	0.9645 (7)
H2	1.1978	−0.0455	0.2159	0.023*	0.9645 (7)
C3	1.38770 (17)	0.07272 (13)	0.24168 (8)	0.0223 (2)	0.9645 (7)
H3	1.4342	0.0227	0.2892	0.027*	0.9645 (7)
C4	1.46102 (14)	0.18583 (13)	0.21639 (8)	0.0219 (3)	0.9645 (7)
H4	1.5567	0.2143	0.2468	0.026*	0.9645 (7)
C5	1.39259 (15)	0.25668 (13)	0.14615 (8)	0.0227 (3)	0.9645 (7)
H5	1.4426	0.3334	0.1276	0.027*	0.9645 (7)
C6	1.25096 (14)	0.21603 (11)	0.10265 (7)	0.0195 (2)	0.9645 (7)
H6	1.2057	0.2662	0.0549	0.023*	0.9645 (7)
C7	0.86874 (13)	0.21393 (10)	0.04130 (7)	0.0164 (2)	0.9645 (7)
C8	0.89979 (14)	0.32572 (11)	0.09251 (7)	0.0204 (2)	0.9645 (7)
H8	0.9785	0.3220	0.1401	0.024*	0.9645 (7)
C9	0.81825 (15)	0.44211 (11)	0.07537 (8)	0.0239 (3)	0.9645 (7)
H9	0.8428	0.5170	0.1105	0.029*	0.9645 (7)
C10	0.70130 (16)	0.44902 (12)	0.00709 (8)	0.0242 (3)	0.9645 (7)
H10	0.6463	0.5287	−0.0051	0.029*	0.9645 (7)
C11	0.66515 (16)	0.33862 (12)	−0.04334 (8)	0.0255 (3)	0.9645 (7)
H11	0.5835	0.3421	−0.0894	0.031*	0.9645 (7)
C12	0.74836 (14)	0.22282 (12)	−0.02654 (8)	0.0215 (2)	0.9645 (7)
H12	0.7230	0.1482	−0.0618	0.026*	0.9645 (7)
C13	0.85261 (14)	−0.04591 (11)	0.13883 (7)	0.0173 (2)	0.9645 (7)
C14	0.85115 (15)	0.02324 (12)	0.22736 (8)	0.0210 (2)	0.9645 (7)
H14A	0.7804	−0.0234	0.2630	0.032*	0.9645 (7)
H14B	0.8147	0.1136	0.2183	0.032*	0.9645 (7)
H14C	0.9574	0.0235	0.2572	0.032*	0.9645 (7)
C15	0.91134 (15)	−0.18758 (12)	0.15272 (8)	0.0218 (3)	0.9645 (7)
H15A	1.0199	−0.1866	0.1794	0.033*	0.9645 (7)
H15B	0.9068	−0.2327	0.0966	0.033*	0.9645 (7)
H15C	0.8448	−0.2336	0.1910	0.033*	0.9645 (7)
C16	0.68191 (15)	−0.05123 (12)	0.09687 (8)	0.0219 (2)	0.9645 (7)
H16A	0.6184	−0.1041	0.1332	0.033*	0.9645 (7)
H16B	0.6797	−0.0908	0.0390	0.033*	0.9645 (7)
H16C	0.6391	0.0377	0.0917	0.033*	0.9645 (7)
Si1A	0.8638 (8)	0.0135 (6)	0.0097 (4)	0.01404 (9)	0.0355 (7)
C1A	0.7401 (14)	−0.0682 (9)	−0.0846 (6)	0.0163 (2)	0.0355 (7)
C2A	0.712 (2)	−0.0099 (12)	−0.1659 (6)	0.0191 (2)	0.0355 (7)
H2A	0.7528	0.0749	−0.1757	0.023*	0.0355 (7)
C3A	0.625 (3)	−0.0757 (16)	−0.2329 (8)	0.0223 (2)	0.0355 (7)
H3A	0.6063	−0.0359	−0.2884	0.027*	0.0355 (7)
C4A	0.566 (3)	−0.1998 (17)	−0.2186 (12)	0.0219 (3)	0.0355 (7)
H4A	0.5065	−0.2448	−0.2643	0.026*	0.0355 (7)
C5A	0.594 (2)	−0.2580 (13)	−0.1373 (12)	0.0227 (3)	0.0355 (7)
H5A	0.5532	−0.3428	−0.1275	0.027*	0.0355 (7)
C6A	0.681 (2)	−0.1922 (9)	−0.0703 (9)	0.0195 (2)	0.0355 (7)
H6A	0.6997	−0.2320	−0.0148	0.023*	0.0355 (7)
C7A	0.8171 (13)	0.1959 (7)	0.0073 (6)	0.0164 (2)	0.0355 (7)
C8A	0.9272 (17)	0.2764 (8)	0.0526 (9)	0.0204 (2)	0.0355 (7)
H8A	1.0252	0.2414	0.0758	0.024*	0.0355 (7)
C9A	0.894 (2)	0.4082 (8)	0.0640 (11)	0.0239 (3)	0.0355 (7)
H9A	0.9694	0.4633	0.0950	0.029*	0.0355 (7)
C10A	0.751 (2)	0.4595 (8)	0.0300 (12)	0.0242 (3)	0.0355 (7)
H10A	0.7282	0.5495	0.0378	0.029*	0.0355 (7)
C11A	0.641 (2)	0.3789 (10)	−0.0153 (13)	0.0255 (3)	0.0355 (7)
H11A	0.5427	0.4139	−0.0385	0.031*	0.0355 (7)
C12A	0.6738 (15)	0.2471 (9)	−0.0267 (9)	0.0215 (2)	0.0355 (7)
H12A	0.5985	0.1920	−0.0577	0.026*	0.0355 (7)
C13A	0.8088 (19)	−0.0519 (11)	0.1193 (6)	0.0173 (2)	0.0355 (7)
C14A	0.871 (3)	0.043 (2)	0.1921 (7)	0.0210 (2)	0.0355 (7)
H14D	0.9855	0.0485	0.1940	0.032*	0.0355 (7)
H14E	0.8253	0.1299	0.1807	0.032*	0.0355 (7)
H14F	0.8417	0.0105	0.2481	0.032*	0.0355 (7)
C15A	0.874 (4)	−0.1902 (16)	0.1401 (13)	0.0218 (3)	0.0355 (7)
H15D	0.8350	−0.2508	0.0940	0.033*	0.0355 (7)
H15E	0.9892	−0.1878	0.1440	0.033*	0.0355 (7)
H15F	0.8404	−0.2198	0.1957	0.033*	0.0355 (7)
C16A	0.6288 (19)	−0.058 (3)	0.1189 (13)	0.0219 (2)	0.0355 (7)
H16D	0.5859	−0.1174	0.0730	0.033*	0.0355 (7)
H16E	0.6013	−0.0892	0.1753	0.033*	0.0355 (7)
H16F	0.5849	0.0301	0.1080	0.033*	0.0355 (7)

### (3) 1,2-Di-tert-butyl-1,1,2,2-tetraphenyldisilane Atomic displacement parameters (Å^2^)

**Table:**

	*U*^11^	*U*^22^	*U*^33^	*U*^12^	*U*^13^	*U*^23^
Si1	0.01699 (16)	0.01075 (15)	0.01390 (15)	0.00070 (11)	−0.00100 (11)	−0.00031 (10)
C1	0.0185 (5)	0.0142 (5)	0.0159 (5)	0.0017 (4)	−0.0001 (4)	−0.0030 (4)
C2	0.0224 (6)	0.0151 (5)	0.0194 (5)	0.0022 (4)	−0.0006 (4)	−0.0014 (4)
C3	0.0226 (6)	0.0213 (5)	0.0217 (6)	0.0056 (5)	−0.0054 (4)	−0.0002 (4)
C4	0.0167 (6)	0.0249 (6)	0.0234 (6)	0.0016 (5)	−0.0022 (4)	−0.0065 (4)
C5	0.0244 (6)	0.0212 (6)	0.0221 (6)	−0.0045 (5)	0.0007 (5)	−0.0025 (4)
C6	0.0230 (6)	0.0175 (5)	0.0175 (5)	−0.0011 (4)	−0.0007 (4)	−0.0010 (4)
C7	0.0197 (5)	0.0135 (5)	0.0159 (5)	0.0014 (4)	0.0005 (4)	0.0004 (4)
C8	0.0249 (6)	0.0146 (5)	0.0207 (5)	0.0026 (4)	−0.0024 (4)	−0.0017 (4)
C9	0.0287 (7)	0.0134 (5)	0.0286 (6)	0.0027 (5)	−0.0016 (5)	−0.0014 (4)
C10	0.0282 (7)	0.0168 (5)	0.0271 (6)	0.0073 (5)	−0.0008 (5)	0.0043 (4)
C11	0.0291 (7)	0.0257 (6)	0.0204 (6)	0.0080 (5)	−0.0053 (5)	0.0011 (5)
C12	0.0243 (6)	0.0201 (6)	0.0193 (5)	0.0046 (5)	−0.0028 (5)	−0.0030 (4)
C13	0.0195 (6)	0.0155 (5)	0.0168 (5)	−0.0004 (4)	0.0009 (4)	−0.0001 (4)
C14	0.0241 (6)	0.0222 (6)	0.0171 (5)	0.0005 (5)	0.0033 (4)	−0.0007 (4)
C15	0.0270 (7)	0.0151 (5)	0.0234 (6)	−0.0016 (5)	0.0028 (5)	0.0024 (4)
C16	0.0201 (6)	0.0235 (6)	0.0223 (6)	−0.0027 (5)	0.0025 (4)	−0.0019 (5)
Si1A	0.01699 (16)	0.01075 (15)	0.01390 (15)	0.00070 (11)	−0.00100 (11)	−0.00031 (10)
C1A	0.0185 (5)	0.0142 (5)	0.0159 (5)	0.0017 (4)	−0.0001 (4)	−0.0030 (4)
C2A	0.0224 (6)	0.0151 (5)	0.0194 (5)	0.0022 (4)	−0.0006 (4)	−0.0014 (4)
C3A	0.0226 (6)	0.0213 (5)	0.0217 (6)	0.0056 (5)	−0.0054 (4)	−0.0002 (4)
C4A	0.0167 (6)	0.0249 (6)	0.0234 (6)	0.0016 (5)	−0.0022 (4)	−0.0065 (4)
C5A	0.0244 (6)	0.0212 (6)	0.0221 (6)	−0.0045 (5)	0.0007 (5)	−0.0025 (4)
C6A	0.0230 (6)	0.0175 (5)	0.0175 (5)	−0.0011 (4)	−0.0007 (4)	−0.0010 (4)
C7A	0.0197 (5)	0.0135 (5)	0.0159 (5)	0.0014 (4)	0.0005 (4)	0.0004 (4)
C8A	0.0249 (6)	0.0146 (5)	0.0207 (5)	0.0026 (4)	−0.0024 (4)	−0.0017 (4)
C9A	0.0287 (7)	0.0134 (5)	0.0286 (6)	0.0027 (5)	−0.0016 (5)	−0.0014 (4)
C10A	0.0282 (7)	0.0168 (5)	0.0271 (6)	0.0073 (5)	−0.0008 (5)	0.0043 (4)
C11A	0.0291 (7)	0.0257 (6)	0.0204 (6)	0.0080 (5)	−0.0053 (5)	0.0011 (5)
C12A	0.0243 (6)	0.0201 (6)	0.0193 (5)	0.0046 (5)	−0.0028 (5)	−0.0030 (4)
C13A	0.0195 (6)	0.0155 (5)	0.0168 (5)	−0.0004 (4)	0.0009 (4)	−0.0001 (4)
C14A	0.0241 (6)	0.0222 (6)	0.0171 (5)	0.0005 (5)	0.0033 (4)	−0.0007 (4)
C15A	0.0270 (7)	0.0151 (5)	0.0234 (6)	−0.0016 (5)	0.0028 (5)	0.0024 (4)
C16A	0.0201 (6)	0.0235 (6)	0.0223 (6)	−0.0027 (5)	0.0025 (4)	−0.0019 (5)

### (3) 1,2-Di-tert-butyl-1,1,2,2-tetraphenyldisilane Geometric parameters (Å, º)

**Table:**

Si1—C7	1.8949 (11)	Si1A—C7A	1.904 (3)
Si1—C1	1.9041 (11)	Si1A—C1A	1.911 (3)
Si1—C13	1.9226 (12)	Si1A—C13A	1.921 (4)
Si1—Si1^i^	2.4002 (6)	Si1A—Si1A^i^	2.396 (14)
C1—C2	1.4020 (14)	C1A—C2A	1.3900
C1—C6	1.4028 (15)	C1A—C6A	1.3900
C2—C3	1.3946 (16)	C2A—C3A	1.3900
C2—H2	0.9500	C2A—H2A	0.9500
C3—C4	1.3884 (16)	C3A—C4A	1.3900
C3—H3	0.9500	C3A—H3A	0.9500
C4—C5	1.3873 (16)	C4A—C5A	1.3900
C4—H4	0.9500	C4A—H4A	0.9500
C5—C6	1.3929 (15)	C5A—C6A	1.3900
C5—H5	0.9500	C5A—H5A	0.9500
C6—H6	0.9500	C6A—H6A	0.9500
C7—C8	1.4000 (14)	C7A—C8A	1.3900
C7—C12	1.4018 (14)	C7A—C12A	1.3900
C8—C9	1.3910 (15)	C8A—C9A	1.3900
C8—H8	0.9500	C8A—H8A	0.9500
C9—C10	1.3860 (16)	C9A—C10A	1.3900
C9—H9	0.9500	C9A—H9A	0.9500
C10—C11	1.3887 (16)	C10A—C11A	1.3900
C10—H10	0.9500	C10A—H10A	0.9500
C11—C12	1.3922 (15)	C11A—C12A	1.3900
C11—H11	0.9500	C11A—H11A	0.9500
C12—H12	0.9500	C12A—H12A	0.9500
C13—C15	1.5398 (16)	C13A—C14A	1.540 (4)
C13—C14	1.5411 (16)	C13A—C16A	1.542 (4)
C13—C16	1.5427 (17)	C13A—C15A	1.542 (4)
C14—H14A	0.9800	C14A—H14D	0.9800
C14—H14B	0.9800	C14A—H14E	0.9800
C14—H14C	0.9800	C14A—H14F	0.9800
C15—H15A	0.9800	C15A—H15D	0.9800
C15—H15B	0.9800	C15A—H15E	0.9800
C15—H15C	0.9800	C15A—H15F	0.9800
C16—H16A	0.9800	C16A—H16D	0.9800
C16—H16B	0.9800	C16A—H16E	0.9800
C16—H16C	0.9800	C16A—H16F	0.9800
			
C7—Si1—C1	105.87 (5)	C7A—Si1A—C1A	108.15 (19)
C7—Si1—C13	106.62 (5)	C7A—Si1A—C13A	106.8 (4)
C1—Si1—C13	111.24 (5)	C1A—Si1A—C13A	110.9 (4)
C7—Si1—Si1^i^	109.90 (4)	C7A—Si1A—Si1A^i^	108.4 (5)
C1—Si1—Si1^i^	110.01 (4)	C1A—Si1A—Si1A^i^	109.3 (5)
C13—Si1—Si1^i^	112.91 (4)	C13A—Si1A—Si1A^i^	113.2 (6)
C2—C1—C6	116.58 (10)	C2A—C1A—C6A	120.0
C2—C1—Si1	123.81 (8)	C2A—C1A—Si1A	122.5 (3)
C6—C1—Si1	119.61 (8)	C6A—C1A—Si1A	117.4 (3)
C3—C2—C1	121.85 (11)	C3A—C2A—C1A	120.0
C3—C2—H2	119.1	C3A—C2A—H2A	120.0
C1—C2—H2	119.1	C1A—C2A—H2A	120.0
C4—C3—C2	120.29 (11)	C2A—C3A—C4A	120.0
C4—C3—H3	119.9	C2A—C3A—H3A	120.0
C2—C3—H3	119.9	C4A—C3A—H3A	120.0
C5—C4—C3	119.05 (11)	C3A—C4A—C5A	120.0
C5—C4—H4	120.5	C3A—C4A—H4A	120.0
C3—C4—H4	120.5	C5A—C4A—H4A	120.0
C4—C5—C6	120.40 (11)	C6A—C5A—C4A	120.0
C4—C5—H5	119.8	C6A—C5A—H5A	120.0
C6—C5—H5	119.8	C4A—C5A—H5A	120.0
C5—C6—C1	121.82 (11)	C5A—C6A—C1A	120.0
C5—C6—H6	119.1	C5A—C6A—H6A	120.0
C1—C6—H6	119.1	C1A—C6A—H6A	120.0
C8—C7—C12	117.19 (10)	C8A—C7A—C12A	120.0
C8—C7—Si1	121.28 (8)	C8A—C7A—Si1A	115.94 (17)
C12—C7—Si1	121.47 (8)	C12A—C7A—Si1A	123.5 (2)
C9—C8—C7	121.60 (10)	C9A—C8A—C7A	120.0
C9—C8—H8	119.2	C9A—C8A—H8A	120.0
C7—C8—H8	119.2	C7A—C8A—H8A	120.0
C10—C9—C8	120.12 (11)	C8A—C9A—C10A	120.0
C10—C9—H9	119.9	C8A—C9A—H9A	120.0
C8—C9—H9	119.9	C10A—C9A—H9A	120.0
C9—C10—C11	119.51 (11)	C11A—C10A—C9A	120.0
C9—C10—H10	120.2	C11A—C10A—H10A	120.0
C11—C10—H10	120.2	C9A—C10A—H10A	120.0
C10—C11—C12	120.12 (11)	C12A—C11A—C10A	120.0
C10—C11—H11	119.9	C12A—C11A—H11A	120.0
C12—C11—H11	119.9	C10A—C11A—H11A	120.0
C11—C12—C7	121.43 (11)	C11A—C12A—C7A	120.0
C11—C12—H12	119.3	C11A—C12A—H12A	120.0
C7—C12—H12	119.3	C7A—C12A—H12A	120.0
C15—C13—C14	109.68 (9)	C14A—C13A—C16A	107.7 (5)
C15—C13—C16	108.02 (9)	C14A—C13A—C15A	109.2 (5)
C14—C13—C16	107.60 (9)	C16A—C13A—C15A	108.0 (5)
C15—C13—Si1	113.05 (8)	C14A—C13A—Si1A	109.2 (5)
C14—C13—Si1	108.90 (8)	C16A—C13A—Si1A	109.7 (5)
C16—C13—Si1	109.45 (8)	C15A—C13A—Si1A	112.8 (5)
C13—C14—H14A	109.5	C13A—C14A—H14D	109.5
C13—C14—H14B	109.5	C13A—C14A—H14E	109.5
H14A—C14—H14B	109.5	H14D—C14A—H14E	109.5
C13—C14—H14C	109.5	C13A—C14A—H14F	109.5
H14A—C14—H14C	109.5	H14D—C14A—H14F	109.5
H14B—C14—H14C	109.5	H14E—C14A—H14F	109.5
C13—C15—H15A	109.5	C13A—C15A—H15D	109.5
C13—C15—H15B	109.5	C13A—C15A—H15E	109.5
H15A—C15—H15B	109.5	H15D—C15A—H15E	109.5
C13—C15—H15C	109.5	C13A—C15A—H15F	109.5
H15A—C15—H15C	109.5	H15D—C15A—H15F	109.5
H15B—C15—H15C	109.5	H15E—C15A—H15F	109.5
C13—C16—H16A	109.5	C13A—C16A—H16D	109.5
C13—C16—H16B	109.5	C13A—C16A—H16E	109.5
H16A—C16—H16B	109.5	H16D—C16A—H16E	109.5
C13—C16—H16C	109.5	C13A—C16A—H16F	109.5
H16A—C16—H16C	109.5	H16D—C16A—H16F	109.5
H16B—C16—H16C	109.5	H16E—C16A—H16F	109.5
			
C6—C1—C2—C3	0.80 (18)	C10—C11—C12—C7	0.5 (2)
Si1—C1—C2—C3	−178.60 (10)	C8—C7—C12—C11	1.18 (18)
C1—C2—C3—C4	−0.1 (2)	Si1—C7—C12—C11	178.14 (10)
C2—C3—C4—C5	−0.9 (2)	C6A—C1A—C2A—C3A	0.0
C3—C4—C5—C6	1.11 (19)	Si1A—C1A—C2A—C3A	178.1 (4)
C4—C5—C6—C1	−0.36 (19)	C1A—C2A—C3A—C4A	0.0
C2—C1—C6—C5	−0.59 (18)	C2A—C3A—C4A—C5A	0.0
Si1—C1—C6—C5	178.83 (10)	C3A—C4A—C5A—C6A	0.0
C1—Si1—C7—C8	−25.36 (11)	C4A—C5A—C6A—C1A	0.0
C13—Si1—C7—C8	93.19 (10)	C2A—C1A—C6A—C5A	0.0
Si1^i^—Si1—C7—C8	−144.13 (9)	Si1A—C1A—C6A—C5A	−178.2 (4)
C1—Si1—C7—C12	157.80 (10)	C12A—C7A—C8A—C9A	0.0
C13—Si1—C7—C12	−83.65 (11)	Si1A—C7A—C8A—C9A	−171.7 (4)
Si1^i^—Si1—C7—C12	39.03 (11)	C7A—C8A—C9A—C10A	0.0
C12—C7—C8—C9	−1.94 (18)	C8A—C9A—C10A—C11A	0.0
Si1—C7—C8—C9	−178.91 (10)	C9A—C10A—C11A—C12A	0.0
C7—C8—C9—C10	1.0 (2)	C10A—C11A—C12A—C7A	0.0
C8—C9—C10—C11	0.7 (2)	C8A—C7A—C12A—C11A	0.0
C9—C10—C11—C12	−1.5 (2)	Si1A—C7A—C12A—C11A	171.0 (4)

Symmetry code: (i) −*x*+2, −*y*, −*z*.

### (3) 1,2-Di-tert-butyl-1,1,2,2-tetraphenyldisilane Hydrogen-bond geometry (Å, º)

Cg1 is the centroid of C1–C6 the ring.

**Table:**

*D*—H···*A*	*D*—H	H···*A*	*D*···*A*	*D*—H···*A*
C15—H15*C*···*Cg*1^ii^	0.98	2.93	3.8955 (14)	171

Symmetry code: (ii) −*x*+2, *y*−1/2, −*z*+1/2.
